# Genetic analysis of flagellar-mediated surface sensing by *Pseudomonas aeruginosa* PA14

**DOI:** 10.1128/jb.00520-24

**Published:** 2025-06-05

**Authors:** Sherry L. Kuchma, C. J. Geiger, Shanice S. Webster, Yu Fu, Robert Montoya, George A. O’Toole

**Affiliations:** 1 Department of Microbiology and Immunology, Geisel School of Medicine at Dartmouth, Hanover, New Hampshire, USA; 2 Howard Hughes Medical Institute 2405, Chevy Chase, Maryland, USA; 3 Department of Biology, Duke University 118713, Durham, North Carolina, USA; University of California San Francisco, San Francisco, California, USA

**Keywords:** surface sensing, flagella, biofilm, stators, *Pseudomonas aeruginosa*

## Abstract

Surface sensing is a key aspect of the early stage of biofilm formation. For *Pseudomonas aeruginosa* PA14, the type IV pili (T4P), the T4P alignment complex, and PilY1 were shown to play a key role in c-di-GMP signaling upon surface contact. The role of the flagellar machinery in surface sensing is less well understood for *P. aeruginosa*. Here, we show, consistent with findings from other groups, that a mutation in the gene encoding the flagellar hook protein (Δ*flgK*) or flagellin (Δ*fliC*) results in a strain that overproduces the Pel exopolysaccharide (EPS) with a concomitant increase in c-di-GMP levels. We use a candidate gene approach and genetic screens, combined with phenotypic assays, to identify key roles for the MotAB and MotCD stators and the FliG protein, a component of the flagellar switch complex, in stimulating the surface-dependent, increased c-di-GMP level noted for these flagellar mutants. These findings are consistent with previous studies showing a role for the stators in surface sensing. We also show that mutations in the genes coding for the DGCs SadC and RoeA, as well as SadB, a protein involved in early surface colonization, abrogate the increased c-d-GMP-related phenotypes of the Δ*flgK* mutant. Together, these data indicate that bacteria monitor the status of flagellar synthesis and function during surface sensing as a mechanism to trigger the biofilm program.

**IMPORTANCE:**

Understanding how the flagellum contributes to surface sensing for *P. aeruginosa* is key to elucidating the mechanisms of biofilm initiation by this important opportunistic pathogen. Here, we take advantage of the observation that mutations in the flagellar hook protein or flagellin enhance surface sensing. We exploit this phenotype to identify key players in this signaling pathway, a critical first step in understanding the mechanistic basis of flagellar-mediated surface sensing. Our findings establish a framework for the future study of flagellar-based surface sensing.

## INTRODUCTION

Bacterial biofilms were first formally described in the 1930s (1), since then, this ubiquitous mode of sessile bacterial growth is important in both medical and industrial settings (2, 3). The first step in the transition from free swimming planktonic cells to the biofilm mode of growth is the microbe contacting the surface and relaying this input signal to the cell to initiate the biofilm mode of growth, a process known as “surface sensing” (4–6).

Many bacteria rely on motility appendages, including flagella and type IV pili, to sense and traverse surfaces. These molecular machines have been shown to be necessary for proper biofilm formation and have been implicated in surface sensing (4–9), but the mechanism(s) by which these appendages sense and transmit the surface sensing signal are just beginning to emerge. Several early studies demonstrated that the bacterial flagellum responds to mechanical load, which, in turn, can serve as a signal of surface engagement. For example, by manipulating the viscosity of the liquid culture or by adding antibodies specific to the flagellum, surface-associated phenotypes were achieved during liquid culture conditions (10–12), indicating that it is the interference in bacterial flagellum function that is the proximal means whereby microbes detect surface engagement.

Bacterial flagella are used to propel the cell body in both liquid and across surfaces (13). A flagellum is composed of a basal-body structure that spans the cellular envelope in bacteria. A hook and flagellar filament extend from the cell body, and upon rotation, propel the cell body forward (14). This molecular machine uses ion motive force, generated by a gradient of protons or sodium ions across the cytoplasmic membrane, to rotate the flagellar filament (15–17). This conversion of chemical potential to flagellar rotation is achieved by stator units that can dynamically bind and dissociate from the flagellar motor (18, 19). Stators are composed of an inner membrane (IM) pentamer and a central dimer unit that plugs the ion pore when stators are not incorporated in the flagellum. Upon incorporation into the flagellum, the inner stator dimer binds the peptidoglycan (PG) layer, unplugging the ion channel within the stator unit, which allows for ion flow down the concentration gradient. This chemical energy is harnessed by the stator units in the form of torque that is transferred to the C-ring of the flagellum via electrostatic interactions with FliG (20, 21). It has been demonstrated that when the flagellar motor experiences a mechanical load, it is able to remodel and recruit additional stator units to aid in rotation (19, 22–24), indicating that changes in external load are sensed by the flagellum, and stator occupancy is a readout for this signal.

Recently, studies using different polar flagellated, monotrichous bacteria have revealed striking similarities in the mechanism by which they use their flagellum to sense surfaces. *Vibrio cholerae*, *Caulobacter crescentus,* and *Pseudomonas aeruginosa* have all been used as model organisms to study flagellar-mediated surface sensing and biofilm initiation. One similarity among these model systems is that mutating genes required for flagellar biosynthesis result in surface-associated phenotypes, including exopolysaccharide (EPS) over-production (25–31). Furthermore, enhanced EPS production was dependent on an increase in the second messenger c-di-GMP, which was often the result of increased level/activity of one or more diguanylate cyclases (DGCs) (25, 28, 29). Finally, the EPS over-producer phenotype exhibited by different flagellar mutants is not universally dependent on stator function (28, 29), as shown by exceptionally thorough analyses of flagellar mutant-associated EPS phenotypes and their stator requirements in *V. cholerae* and *C. crescentus* (28, 29). In general, flagellar mutants defective in early stages of flagellar biosynthesis, that is, steps that disrupt assembly of the basal body and motor structures, lack a stator requirement for EPS overproduction. By contrast, mutants defective in late stages of flagellar assembly, such as those steps predicted to assemble a basal body and motor but lack a flagellar filament, did require stators for these phenotypes. These observations link the stators to flagellar-mediated surface sensing, particularly when the flagellar machine is almost completely assembled.

While there are similarities in flagellar-mediated surface sensing between *V. cholerae*, *C. crescentus*, and *P. aeruginosa* as described above, the flagellar motor of *P. aeruginosa* is notably distinct among these microbes in that it can accommodate two different sets of stators, MotAB and MotCD. Moreover, these two sets of stators have distinct roles in surface motility: MotAB is necessary for maximum velocity during swimming motility, whereas MotCD is absolutely required for swarming motility (32–35). In addition, the MotCD stator has also been shown to be directly involved in surface sensing by binding to the diguanylate cyclase (DGC) SadC and stimulating c-di-GMP production upon surface contact (36). This interaction is mediated by the c-di-GMP-binding protein FlgZ when it is bound to c-di-GMP. The FlgZ·c-di-GMP complex is required for the removal of MotCD stator units from the flagellar motor, leading to shutdown of flagellar rotation while stimulating c-di-GMP production (37). These data indicate that the flagellum, stators, and SadC are important for surface sensing, but there remain missing links in how these complexes are coordinated upon surface contact. The dual-stator system of *P. aeruginosa* may offer unique insights into how bacteria optimize motility and signaling in response to surface engagement.

While proteins involved in flagellum-mediated surface sensing by *P. aeruginosa* have been identified, the mechanism whereby c-di-GMP is increased after initial surface contact by the cell remains largely a mystery. Here, we use a combination of genetic screens and candidate gene studies, combined with phenotypic assays, to begin to investigate how *P. aeruginosa* uses its flagellum to sense a surface.

## RESULTS

### Mutations in the *flgK* and *fliC* genes result in a Pel-dependent increase in Congo red binding and wrinkly colony morphology

**Fig 1:**
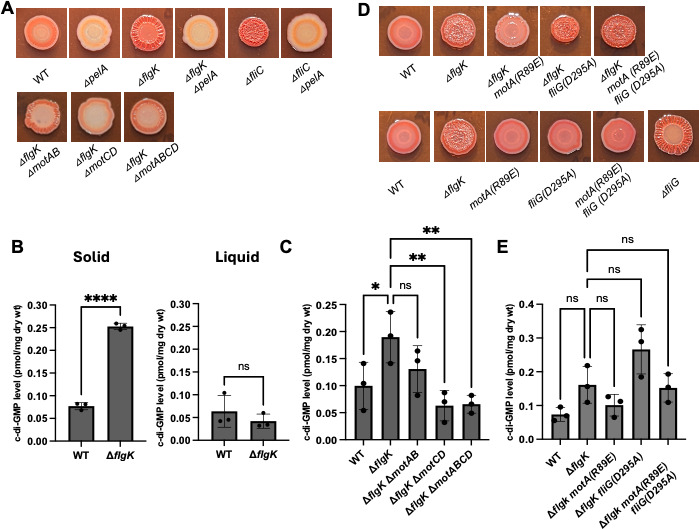
Mutations that eliminate stator production or impact stator occupancy suppress the Δ*flgK* mutant hyper-signaling phenotype. (A and D) Representative CR images of the indicated strains cultured on M8 plates solidified with 1% agar for 16 h at 37°C, followed by 4 days at room temperature. (B) Quantification of c-di-GMP levels in the indicated strains grown either on M8 swarm agar plates (left) or in M8 liquid (right) for extraction of nucleotides and measurement of c-di-GMP levels via mass spectrometry. (C and E) Quantification of c-di-GMP levels in the indicated strains grown on M8 agar swarm plates for 16 h prior to harvest for extraction of nucleotides and measurement of c-di-GMP levels via mass spectrometry. Experiments (B, C, and E) were performed in triplicate with three technical replicates per strain. Data in experiment in (B) were analyzed using an unpaired t-test. Data in experiments (C and E) were analyzed by ANOVA with Dunnett’s multiple comparisons test. Significant differences are shown for comparisons to the Δ*flgK* strain. ns, non-significant difference; *P < 0.05 and **P < 0.01.

A recent publication that included members of our team demonstrated that mutating the gene encoding the hook-associated protein FlgK or the gene encoding the flagellin FliC of *P. aeruginosa* PA14 led to an increase in the production of the Pel EPS by those mutant strains (31). A similar observation was previously made by Parsek and colleagues for *P. aeruginosa* PAO1 when characterizing rugose small colony variants (RSCVs) isolated from biofilm-grown populations and cystic fibrosis (CF) sputum isolates (30). When plated on Congo Red (CR) agar, the *P. aeruginosa* PA14 Δ*flgK* and Δ*fliC* mutants showed enhanced binding of the dye CR and a wrinkly colony morphology; these phenotypes were dependent on production of the Pel polysaccharide (38–40) (Fig. 1A, top row).

### Identification of factors required for the Congo red phenotypes of the Δ*flgK* mutant using a genetic screen

To gain insight into why the Δf*lgK* mutant exhibited enhanced Pel production, we performed transposon mutagenesis of the Δ*flgK* mutant, then plated the mutants on CR medium to evaluate Pel production and colony morphology. We screened approximately ~10,000 mutants and identified insertions in 44 genes. In Table 1, we list a subset of the mutants with transposon insertions mapping to genes with known links to Pel production and/or c-di-GMP signaling pathways. The majority of these mutants exhibited suppression of the Δ*flgK* mutant CR phenotype. For example, numerous transposon insertions mapped to genes required for Pel biosynthesis and secretion machinery, as expected, which served to validate the screen. We also identified mutations mapping to genes required for c-di-GMP production, including the DGC-encoding *roeA* gene, again validating the screen. Mutants exhibiting enhancement of the CR phenotype of the Δ*flgK* mutant were also isolated with mutations mapping to genes shown to be involved in biofilm formation, such as *retS* and *bifA* (Fig. S1A) (41, 42). The *retS* gene encodes a hybrid sensor kinase/response regulator shown to be involved in activation of virulence gene expression and repression of EPS production in *P. aeruginosa* strain PAO1, and a *retS* mutant exhibits hyper-biofilm formation (41). Similarly, the *bifA* gene encodes a c-di-GMP-degrading phosphodiesterase (PDE), and a Δ*bifA* mutant showed enhanced CR binding, wrinkly colony morphology, and hyper-biofilm formation that is both Pel- and c-di-GMP-dependent (42, 43). Therefore, isolation of mutations in *bifA* and *retS* as enhancers of Δ*flgK* CR phenotypes is consistent with their reported roles in repressing EPS production.

**TABLE 1:** Mutations identified from Congo red transposon screen in *flgK* mutant background with known links to EPS production and/or surface signaling

Candidate^*a*^	Predicted or known function encoded by the gene	Congo red phenotype^*b*^	Number of alleles isolated
Pel biosynthetic operon			
*pelB*	Forms part of the Pel secretion complex	Reduced	1
*pelD*	cdG binding protein; important for Pel secretion	Reduced	1
*pelA*	Component of the Pel secretion complex	Reduced	2
*pelE*	Component of the Pel secretion complex	Reduced	2
*pelC*	Component of the Pel secretion complex	Reduced	1
*pelF*	Component of the Pel secretion complex	Reduced	1
*pelG*	Component of the Pel secretion complex	Reduced	3
T4P-related functions			
*pilQ*	T4P secretin protein	Reduced	1
*pilW*	Minor pilin, forms the T4P assembly	Reduced	1
*pilY1*	Important in pili assembly and mechanosensing	Reduced	7
*pilV*	Minor pilin	Reduced	2
*pilX*	Minor pilin	Reduced	1
c-di-GMP-related functions			
*roeA*	A diguanylate cyclase (DGC)	Reduced	1
*retS*	Regulator of c-di-GMP level, EPS, T3SS	Enhanced	1
*bifA-sodB*	Intergenic transposon insertion between *bifA* (phosphodiesterase [PDE]) and *sodB* (superoxide dismutase)	Enhanced	1
*pvrS*	PvrS part of a two-component system with PvrR (a PDE)	Reduced	9^*c*^

^*a*^ The source of the gene information is Pseudomonas.com.

^*b*^ Reduced CR phenotype indicates reduction in both red color intensity and wrinkled colony morphology, unless otherwise indicated, whereas enhanced phenotype refers to an increase in both color and wrinkling.

^*c*^ Alleles of *pvrS* were frequently isolated as suppressors of enhanced CR due to elevated c-di-GMP levels in a previously published screen; these alleles were shown to result in the overexpression of the adjacent *pvrR* gene encoding a PDE.

An additional class of mutants identified in the screen impacted genes involved in type IV pili function, with known roles in second messenger signaling (44–49). Transposon insertions that map to the *pilW* and *pilY1* genes (arranged in an operon consisting of the *fimUpilWXY1E* genes) strongly suppress the Δ*flgK* CR phenotypes (Fig. S1B). These results are consistent with previous findings from our group showing that *pilY1*, *pilW,* and *pilX* participate not only in type IV pili biogenesis but also in surface signaling (43–45, 48). A Δ*flgK* Δ*pilY1* double mutant shows reduced CR binding and wrinkling compared to the Δ*flgK* mutant, but the suppression is not as strong as observed for either the Δ*flgK pilY1*::Tn or Δ*flgK pilW*::Tn mutants, suggesting the transposon insertions likely impact the function of the entire operon and that there is some functional redundancy among these genes in the Δ*flgK* strain background. We further address the implications of these findings supporting a relationship between T4P and flagellar signaling in the Discussion.

Finally, we isolated a subset of mutants with transposon insertions in uncharacterized genes or those with no obvious or previously explored connection to EPS production or c-di-GMP signaling pathways, and those are listed in Table S1. This set of mutants includes those that exhibit enhancement (18 mutations) or reduction (12 mutations) of the Δ*flgK* CR phenotypes. Further work is needed to characterize whether and what role these genes may play in impacting EPS production, c-di-GMP metabolism, or other aspects of surface sensing and signaling.

### The increase in c-di-GMP levels for the Δ*flgK* mutant requires surface growth

The increased CR binding and wrinkly colony morphology have been associated with increased c-di-GMP levels for *P. aeruginosa* strains and in other organisms with mutations in their flagellar machinery. To assess whether the Δ*flgK* mutant accumulated increased c-di-GMP when grown specifically on a surface, we quantified c-di-GMP levels of surface-grown cells using mass spectrometry and observed that the Δ*flgK* mutant showed a significant increase in c-di-GMP levels relative to the WT (Fig. 1B, left). By contrast, the liquid-grown Δ*flgK* mutant showed a non-significant change in c-di-GMP levels relative to the WT (Fig. 1B, right). These data suggest that the increased levels of c-di-GMP in the Δ*flgK* mutant require surface growth, findings that are consistent with the aforementioned study showing a surface-contact-dependent induction of Pel and Psl polysaccharides by flagellar mutants of *P. aeruginosa* strain PAO1 (30).

### The stators are required for the Pel-dependent increase in Congo red binding and wrinkly colony morphology of the Δf*lgK* mutant

Previous studies from our group have shown that the stators play a key role in surface sensing and modulating c-di-GMP levels (32, 35, 36, 50). As previously described in other bacteria, a wrinkly colony/EPS over-producer phenotype of mutants defective in later stages of flagellar synthesis was dependent on the presence of functional stator units (28–30). Given that the flagellar hook-associated protein FlgK is required for later stages of flagellar biosynthesis and Δ*flgK* mutants are expected to assemble a basal body structure, we predicted that enhanced signaling in this mutant would require functional stators. To test this hypothesis, we mutated one or both stator sets in the Δ*flgK* mutant background. Deletion of either set of stators reduced the amount of CR binding and the wrinkled colony phenotype to a similar degree relative to the Δ*flgK* mutant alone (Fig. 1A, bottom row).

We next asked whether the loss of CR binding and wrinkly colony morphology for the Δ*flgK* mutant carrying the stator mutants was associated with reduced c-di-GMP levels. As shown in Fig. 1C, the Δ*flgK*Δ*motCD* mutant exhibited a ~3-fold reduction in c-di-GMP levels relative to the Δ*flgK* parent, whereas the Δ*flgK*Δ*motAB* mutant showed a modest but non-significant reduction in c-di-GMP levels. Deletion of both stator sets in the Δ*flgK* mutant strain (Δ*flgK*Δ*motAB*Δ*motCD*) showed a significant reduction in c-di-GMP levels comparable to the Δ*flgK*Δ*motCD* strain. Taken together, these data indicate that the stators are required for the enhanced EPS production by the Δ*flgK* mutant and that the MotCD stator set may play a more pronounced role in influencing c-di-GMP levels than the MotAB stator set in the context of the Δ*flgK* mutant, an observation that is consistent with our previous findings (36).

### Mutations in the switch complex impact Congo red binding and wrinkly colony morphology of the Δ*flgK* mutant

The interaction of the cytoplasmic portion of MotA with the FliG protein, a member of the rotor (aka, the switch complex, named for its role in switching the direction of rotation of the motor) is thought to be important for stator incorporation into the flagellar motor. Studies in *E. coli* and *Salmonella* have identified key residues in MotA and FliG that are involved in electrostatic interactions between these proteins, which are thought to be critical sites of contact enabling stator incorporation (20, 21, 51). For example, an R90E charge reversal mutation in MotA of *E. coli* led to loss of motility similar to a *motA* deletion, and furthermore, this motility defect was partially rescued by amino acid substitutions that reversed or neutralized the charge of the FliG-D289 residue (21).

Based on the findings in *E. coli*, we introduced the analogous *motA* mutation (R89E) onto the chromosome of *P. aeruginosa* in the native *motA* locus of the WT and the Δ*flgK* strains to ask whether MotA-FliG interactions are required for enhanced Pel production in the Δ*flgK* mutant (see Fig. S2A, MotA protein alignment). We found that the MotA-R89E mutant protein markedly reduced CR binding and colony wrinkling of the Δ*flgK* mutant (Fig. 1D, top row), with no obvious impact on these phenotypes in the WT background (Fig. 1D, bottom row). The R89E mutation also led to a decrease in c-di-GMP levels in the Δ*flgK* strain, but this effect was not significantly different when controlling for multiple comparisons (Fig. 1E). Notably, the R89E mutation has no detectable impact on MotA protein levels, as previously shown (35) (see protein alignment methods section for additional information), discounting the possibility that reduced protein stability impacts these phenotypes.

Next, we introduced a mutation in *fliG* (FliG-D295A) into the WT and Δ*flgK* mutant strains. The D295A mutation is analogous to the D289A substitution of *E. coli,* which rescued the motility defect of the strain expressing the MotA(R90E) mutant protein (Fig. S2B, FliG alignment). We selected the D295A negative charge to neutral substitution to reduce the likelihood that this mutation would also impact electrostatic interactions between FliG and the MotCD stator, which could complicate the analysis. Interestingly, the strain with FliG-D295A mutant protein led to increased CR binding and wrinkling compared to the Δ*flgK* background alone, with no impact on these phenotypes in the WT strain (Fig. 1D and E). The *fliG* deletion strain (Fig. 1D, bottom row, right), which exhibits hyper-CR binding (a phenotype we revisit below), is included as a control, confirming that the *fliG*(D295A) mutation is distinct from a null mutant. As expected, given the CR phenotype, the Δ*flgK fliG*(D295A) strain exhibited higher c-di-GMP levels relative to the Δ*flgK* mutant alone, but the increase was not statistically significant after controlling for multiple comparisons.

We then generated the *fliG*(D295A) *motA*(R89E) Δ*flgK* triple mutant strain and observed an intermediate phenotype compared to the double mutant strains, which essentially restored the Δ*flgK* single mutant CR binding and wrinkly colony appearance. As with the single *motA* (R89E) and *fliG* (D295A) mutants, the double *motA* (R98E) *fliG* (D289A) mutant did not exhibit changes in CR binding or colony morphology in the WT background, indicating that the changes in these phenotypes are specific to the Δ*flgK* background (Fig. 1D and E). Overall, interactions between the stator and switch complex appear to have a modest impact on c-di-GMP levels, in contrast to the magnitude of change observed for deletion of the stators, but still resulted in visible changes to CR binding.

### Mutations that prevent proton binding suppress the Congo red binding, wrinkly colony phenotype, and increased c-di-GMP levels in the Δ*flgK* background

Stators generate torque via ion flux through the IM channel formed by the MotAB stator complex when bound to the motor. In *E. coli*, the MotB-D32 residue is considered critical for proton binding and flux through the stator channel (52). As such, a D32A mutation renders MotB unable to bind protons (52), which results in loss of motor occupancy (19). Studies in *Salmonella* showed that MotB-D33N mutant stators were able to associate with the motor but exhibited an increased rate of dissociation relative to wild-type MotAB stators (20, 53). Together, these studies indicate that proton binding and/or transport is important for stator incorporation and/or stability in the motor, and thus stator function.

**Fig 2:**
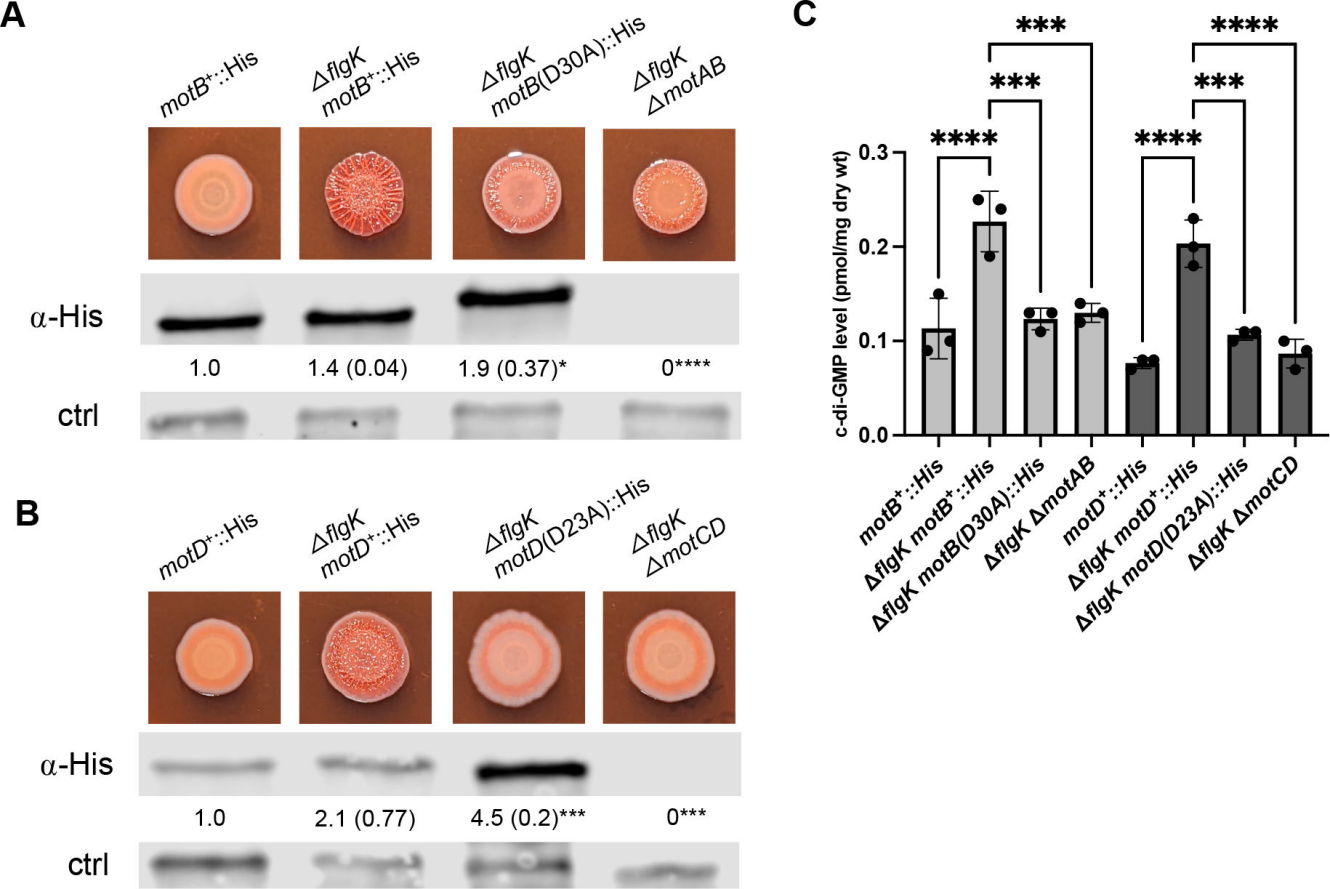
Mutations that impact proton binding suppress hyper-signaling. (**A**) The top panel shows representative CR images of the indicated strains. The proton-binding aspartate residue MotB-D30, analogous to the *E. coli* MotB-D32, allele is mutated to alanine in the Δ*flgK* deletion strain. The middle panel shows the Western blot for the MotB-His_6_ WT and D30A variant epitope-tagged proteins detected in lysate samples from surface-grown strains using an anti-His antibody (α-His). MotB-His_6_ protein levels were quantified and normalized to a cross-reacting band (bottom panel, ctrl) detected in all samples and used as a loading control. Numbers below the middle panel show the mean and standard deviation (SD), in parentheses, from three independent experiments, normalized to the WT, which is set to 1.0. Statistical analysis was performed using ANOVA with Dunnett’s test for multiple comparisons. Significant differences are shown for comparisons to the Δ*flgK motB*^+^-His_6_ strain, with *P < 0.05 and ****P < 0.0001. (**B**). The top panel shows representative CR images of the indicated strains. The proton-binding aspartate residue of MotD-D23 is mutated to alanine in the Δ*flgK* deletion strain. The middle panel shows the MotD WT and D23A His_6_-epitope tagged proteins detected and quantified as described in panel A. Significant differences are shown for comparisons to the Δ*flgK motD*^+^-His_6_ strain. *P < 0.05; ***P < 0.0005. (**C**). Quantification of c-di-GMP for the indicated strains grown on M8 swarm plates for 16 h. Experiments were performed in triplicate with three technical replicates per strain and analyzed by ANOVA with Tukey’s post-test comparison. Significant differences are shown for comparisons either to the Δ*flgK motB*^+^::His strain or to the Δ*flgK motD*^+^::His strain as indicated. ns, non-significant difference; significant differences noted as follows: ***P < 0.001; ****P < 0.0001.

To test whether proton-binding ability impacts Δ*flgK* signaling, we constructed the analogous mutation to *E. coli* MotB-D32A in the *P. aeruginosa motB* and *motD* genes (resulting in the amino acid changes D30A and D23A, respectively; see Fig. S2C for a MotB/D protein alignment) and introduced these mutations onto the chromosome at their native loci. We first confirmed that these mutations did not negatively impact protein levels by performing Western blots to detect the His_6_ epitope-tagged MotB or MotD WT and mutant variants (Fig. 2A and B, middle panels). In fact, the MotB-D30A and MotD-D23A proteins are detected at relatively higher abundance compared to their WT counterparts. We also observed that the MotB-D30A variant protein migrated more slowly than the WT protein in the SDS-polyacrylamide gel (Fig. 2A). This difference in migration is not due to a mutation in the coding sequence, as confirmed by PCR and sequencing of genomic DNA from the *motB* locus in the WT and MotB-D30A strains (see Materials and Methods for details). Notably, MotB-D32 variants have been shown to exhibit altered mobility in SDS-polyacrylamide gels and increased abundance relative to WT MotB in *E. coli* (52).

Stator proteins are believed to reside as heptameric [MotA(or C)_5_/MotB(or D)_2_] complexes in inner membrane pools, available for rapid incorporation into the flagellar motor (54, 55). We performed cellular fractionations to ascertain whether these variants localize to the cell membrane in the same manner as the WT proteins. While we again observed similar changes in MotB-D30A mobility and MotD-D23A abundance, consistent with results in Fig. 2A and B, the MotB and MotD variants were localized to the total membrane (TM) fraction as expected, with no obvious aberrant accumulation in the cytoplasm (Cyt) relative to their WT counterparts (Fig. S3). A WT strain expressing a cytoplasmic-localized GFP protein served as a control for the fractionation method to validate the separation of the cytoplasmic and total membrane fractions. Indeed, the GFP protein is largely in the whole cell and cytoplasmic fractions with minimal cross-reactivity in the total membrane fraction, confirming our assessments of MotB and MotD localization.

We next assessed motility phenotypes of these point mutants using soft agar swim and swarm assays. Given the critical role of the aspartate residue in proton conductance and stator function, as well as the importance of *motCD* in powering soft agar-based motility, we expected that the *motD*(D23A) mutant strain would phenocopy the Δ*motD* mutant in motility assays. This is indeed what we observe, as shown in Fig. S4A; both the deletion and point mutants are defective for swimming and swarming motility.

The data are more nuanced for MotB-D30A. We have previously shown that the MotAB stator promotes swimming motility with MotCD but negatively impacts swarming, likely by competing for motor incorporation with MotCD, which is absolutely required for this form of motility. Thus, Δ*motB* and Δ*motAB* strains show reduced swimming and enhanced swarming relative to WT (Fig. S4B). For the strain carrying the MotB-D30A variant, swimming is further reduced relative to the Δ*motB* and Δ*motAB* mutants, and swarming is comparable to the WT. We interpret these findings to indicate that the MotB-D30A variant may exert a dominant negative impact on motility, possibly by competing with MotCD for motor incorporation, leading to reduced motility. Similarly, *E. coli* and *Salmonella* MotB proton-binding variants displayed dominant negative effects on swimming motility when expressed in the presence of WT MotB counterparts (52, 53).

Finally, we assessed the CR-binding phenotypes and found that both MotB and MotD point mutations in the aspartate residue required for ion flux phenocopied the deletion mutants of the respective stator sets for both CR binding, wrinkly colony morphology, and c-di-GMP levels (Fig. 2A through C), indicating that the proton binding, likely via stator occupancy of MotB and MotD, is important for their role in increased signaling by the Δ*flgK* mutant. These findings are consistent with recent studies in *V. cholerae* and *C. crescentus* showing that sodium ion or proton binding aspartate mutations, respectively, abolished elevated EPS production by flagellar mutants in these microbes (28, 29). Taken together with the switch complex data above, these data are consistent with the previous findings that stator occupancy in the motor is required for signaling.

### The DGCs SadC and RoeA are required for the enhanced c-di-GMP levels in the Δ*flgK* mutant

**Fig 3:**
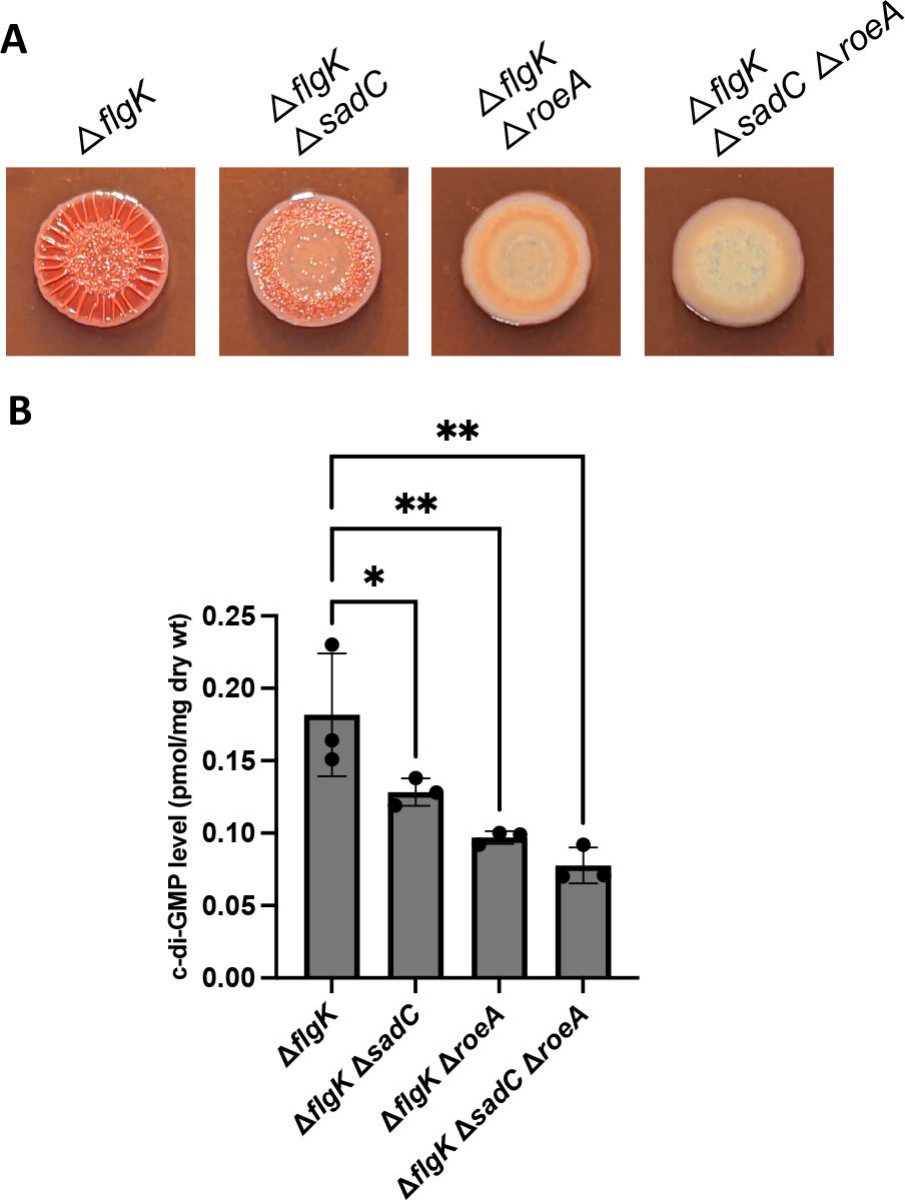
The DGCs SadC and RoeA are required for hyper-signaling in the *flgK* mutant. (**A**) Representative CR images of the indicated strains. (**B**) Quantification of c-di-GMP extracted from swarm-grown strains. Experiments were performed in triplicate with three technical replicates per strain and analyzed by ANOVA with Dunnett’s post-test comparison. Significant differences are shown for comparisons to the Δ*flgK* mutant; *P < 0.05, **P < 0.005.

Previous studies from our team have implicated the SadC and RoeA DGCs as key for early biofilm formation and surface sensing (36, 43, 46, 49, 56, 57). As noted above, a transposon mutation in the *roeA* gene was isolated in the Δ*flgK* CR screen as a suppressor of the enhanced CR-binding phenotype. Therefore, we asked whether null mutations in the *sadC* or *roeA* genes could impact the CR, wrinkly morphology, or c-di-GMP levels of the Δ*flgK* mutant. Mutating the *sadC* or *roeA* genes individually reduced the CR and wrinkly morphology phenotypes of the Δ*flgK* mutant (Fig. 3A), with the *roeA* mutation having a stronger impact on both phenotypes. However, both mutations led to significant reductions in c-di-GMP levels relative to the Δ*flgK* mutant (Fig. 3B). The triple Δ*flgK*Δ*sadC*Δ*roeA* mutant showed a further reduction in CR binding and wrinkly colony phenotypes compared to each of the double mutants, although the change is modest compared to the Δ*flgK* Δ*roeA* mutant (Fig. 3A and B). Together, these data indicate that the c-di-GMP produced in the Δ*flgK* mutant is largely contributed by RoeA and SadC.

### Testing candidate genes for their impact on the phenotypes of the Δ*flgK* mutant

**Fig 4:**
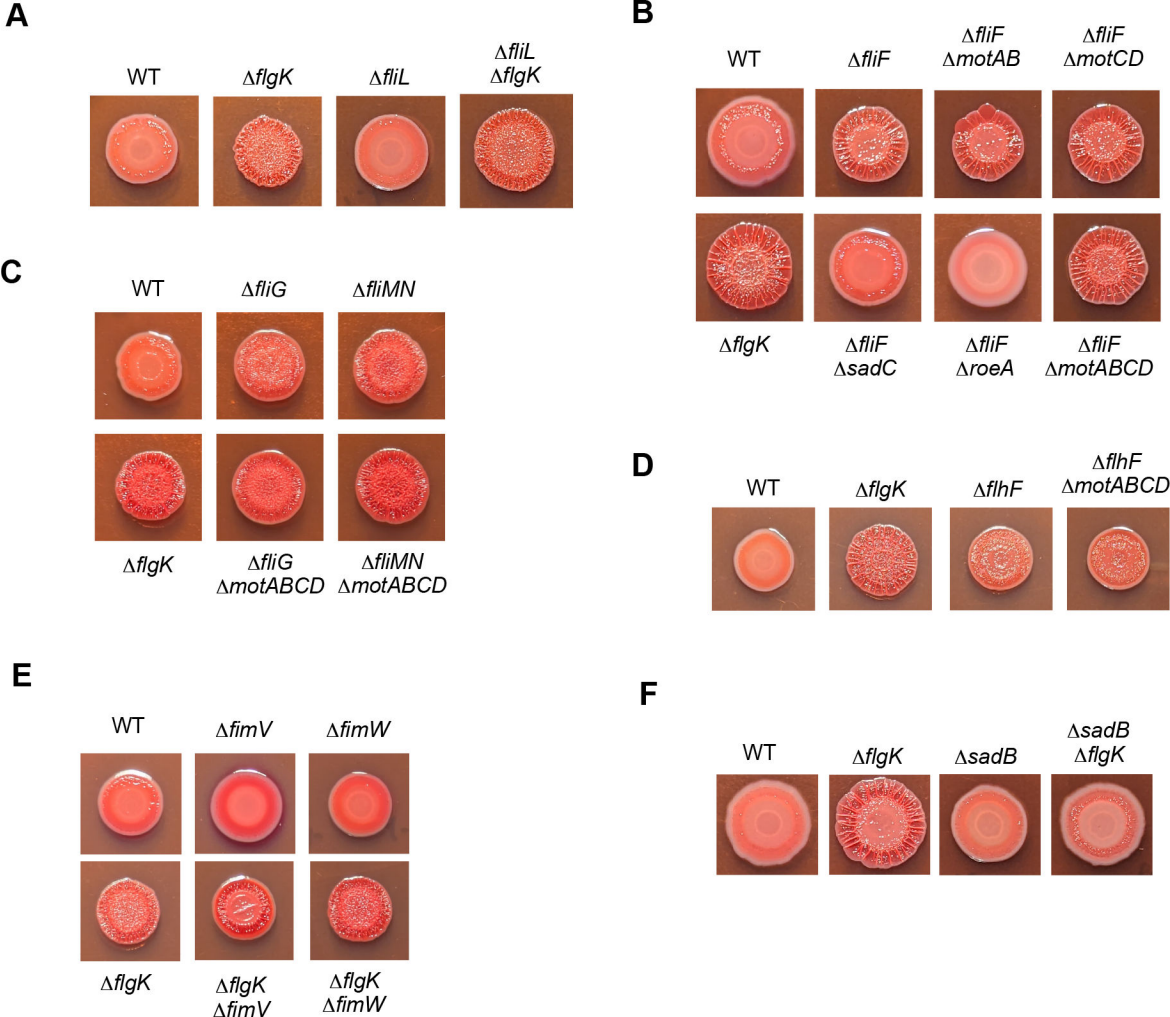
Testing candidate genes for their impact on the Δ*flgK* mutant hyper-signaling phenotype. (A). Representative CR plate images of (**A**) the Δ*fliL* mutation in the WT and the Δ*flgK* mutant background; (**B**) impact of stator mutations, and *sadC* and *roeA* DGC mutations on the Δ*fliF* mutant phenotypes; (**C**) mutations in the *fliG* and *fliMN* genes and impact of the Δ*motABCD* mutation in these mutant backgrounds; (**D**) the Δ*flhF* mutant and impact of the Δ*motABCD* mutation in this mutant; (**E**) mutations in the *fimV* and *fimW* genes in the WT and Δ*flgK* mutant backgrounds; and (**F**) mutation of *sadB* in the WT and Δ*flgK* mutant backgrounds. All CR assays were performed on M8 medium solidified with 1% agar and incubated for 16 h at 37°C followed by an additional 4 days at room temperature.

In addition to the screens described above, we took a candidate gene approach to identify additional genetic factors that may contribute to the Δ*flgK* mutant phenotypes (Fig. 4). For example, we assessed the role of FliL, an accessory protein with a myriad of supporting roles in flagellar assembly/function depending on the microbe (16, 18, 58, 59). The precise function of FliL in *P. aeruginosa* is not fully understood, but it has recently been shown to interact with the stators and influence motile behaviors in liquid environments (59). First, we evaluated motility phenotypes of a Δ*fliL* single mutant using soft agar swim and swarm assays. Δ*fliL* mutants exhibited a modest reduction in swimming, consistent with recent data (59), as well as swarming motility compared to the WT strain (Fig. S5). In CR assays, the Δ*fliL* mutant did not exhibit differences in CR binding or colony phenotypes with respect to the WT, indicating this mutation does not impact Pel production (Fig. 4A). Importantly, mutation of *fliL* did not alter the Δ*flgK* mutant CR phenotypes (Fig. 4A), indicating FliL is not required for enhanced CR binding and wrinkly colony morphology.

By contrast, mutating the *fliF* gene, which encodes the protein comprising the MS ring (membrane-supra-membrane) of the flagellar basal body (58), led to an increase in CR binding and colony wrinkling in the WT background (Fig. 4B). Mutation of *fliF* is expected to disrupt the basal body structure and preclude motor assembly and stator incorporation. Thus, the Δ*fliF* mutant falls into a class of flagellar mutants that impact early stages of flagellar biosynthesis and exhibit enhanced EPS production in a stator-independent manner in other microbes (28, 29). Based on those findings, we assessed whether the stators were required and found that the Δ*fliF* mutant phenotypes are indeed stator-independent, as mutations in *motAB* or *motCD,* as well as in both stator sets (Δ*motABCD*), did not alter the *fliF* enhanced CR binding and colony wrinkling phenotypes (Fig. 4B).

Given this difference between the Δ*fliF* and Δ*flgK* mutants in stator dependence for enhanced signaling, we next tested whether the Δ*fliF* phenotypes are dependent on the SadC and RoeA DGCs, as is the case for the Δ*flgK* mutant, and observed a similar pattern of suppression of the CR phenotype by *sadC* and *roeA* mutations as seen for the Δ*flgK* mutant (Fig. 4B). That is, mutation of *roeA* in the Δ*fliF* background shows stronger suppression of the CR binding and wrinkling than does mutation of *sadC*. These results indicate that these DGCs also contribute to enhanced signaling in a stator-independent pathway, consistent with recent findings from *V. cholerae* (28).

We next assessed the impact of deleting the *fliG* and *fliMN* genes in the WT strain background. Mutating the gene coding for FliG, the rotor component that interacts with the stator complex, or FliMN proteins, which, together with FliG, make up the switch complex to control the direction of flagellar rotation (60), results in phenotypes similar to the Δ*flgK* mutant (Fig. 4C).

The enhanced CR binding and wrinkled colony phenotypes we observe for the Δ*fliG* and Δ*fliMN* mutant phenotypes are consistent with those previously reported for these mutants in other microbes, and like the mutation of the *fliF* gene, these mutations were shown to trigger stator-independent signaling (28, 29). Our results similarly show that the stators are not required for the enhanced signaling in the Δ*fliG* and Δ*fliMN* mutants, as the *motABCD* deletion has no impact on these phenotypes (Fig. 4C).

As a final flagellum biosynthesis factor, we evaluated the role of the FlhF protein in flagellar-mediated hyper-signaling. FlhF is required for positioning of the flagellum at the cell pole, and Δ*flhF* mutant cells exhibit flagella at random sites on the cell body and are impaired for motility (61, 62). More recent studies indicate that FlhF participates in signal transduction pathways that impact surface sensing in *P. aeruginosa* strains PAO1 and PAK (63, 64). We first assessed single Δ*flhF* mutants in motility plate assays and observed a severe defect in swimming motility and lack of swarming motility compared to WT (Fig. S5), consistent with previous findings (62). In CR assays, single Δ*flhF* mutants showed an increase in CR binding and colony wrinkling relative to the WT, in agreement with recent data (63), but these phenotypes were less robust than those observed for the Δ*flgk* mutant (Fig. 4D). We next generated a Δ*motABCD* Δ*flhF* strain to assess whether or not these CR plate phenotypes were stator-dependent. Our results indicate that the stators are not required for the enhanced CR binding and wrinkling observed for the Δ*flhF* mutant (Fig. 4D), suggesting perhaps that polar localization of the flagellar apparatus is important for stator-dependent signaling. However, it is unclear whether the non-polar flagella of the Δ*flhF* mutant are structurally comparable to the WT; thus, further investigation is needed to explore this possibility.

The CR genetic screen described above (Table 1) identified factors required for the Δ*flgK* mutant hyper-signaling phenotype that are related to production/function of T4P and previously shown to be involved in surface sensing, such as PilY1 (45, 48) and PilW (44) (see Fig. S1B). To further assess the roles of additional T4P-related genes with reported roles in c-di-GMP-related signaling, we tested mutations in the *fimV* gene encoding a T4P-associated peptidoglycan binding protein shown to interact with and influence the activity of the DgcP diguanylate cyclase, in addition to involvement in other surface signaling pathways (64–68) and the *fimW* gene encoding a c-di-GMP receptor shown to be involved in early cell-surface commitment (69). We first assessed T4P function using standard twitching motility plate-based assays and found that Δ*fimV* mutants are completely defective, whereas Δ*fimW* mutants are comparable to the WT (not shown), consistent with previously reported results (65, 69). In CR assays, our results show that neither mutation of *fimV* nor *fimW* impacted the Δ*flgK* CR phenotypes (Fig. 4E), indicating these factors are not required for enhanced signaling in the Δ*flgK* mutant, nor did single mutants display elevated CR relative to WT. The Δ*flgK* Δ*fimV* double mutant reproducibly exhibited a smaller colony size on CR plates relative to either single mutant for reasons that are not clear. This phenotype does not appear to be due to loss of T4P function, as we do not observe the same phenotype for other T4P mutants in the Δ*flgK* strain, such as *pilY1* (Fig. S1) or *pilA* (data not shown).

Finally, we assessed whether SadB, a protein required for the transition of cells from reversible to irreversible attachment during early stages of surface association, is necessary for the Δ*flgK* mutant phenotypes. Loss of SadB results in enhanced motility, loss of biofilm formation, and suppression of the hyper-biofilm and Pel-mediated wrinkly morphology phenotype associated with loss of the BifA phosphodiesterase (42, 70, 71). Here we found that the Δ*flgK* Δ*sadB* double mutant reversed the CR-binding and wrinkly phenotypes of the Δ*flgK* mutant (Fig. 4F), indicating that *sadB* is required for enhanced signaling by the Δ*flgK* mutant. These data agree with previous findings showing that *sadB* mutations suppress the RSCV phenotypes of *fliC* mutants (30). While the precise function of SadB is not yet known, the data here that SadB contributes to the flagellum-mediated surface signaling pathway are consistent with its role as an important player in the inverse regulation of motility and biofilm formation (71).

## DISCUSSION

In this study, we explored the observation that a mutation in the *flgK* gene results in an increase in c-di-GMP levels as well as an increase in CR staining and a wrinkly colony morphology. The observation that the increase in c-di-GMP levels in the Δ*flgK* mutant only occurs on a surface suggested a link to a surface-sensing pathway. To explore the link to surface sensing by leveraging the Δ*flgK* mutant, we performed a genetic screen that identified multiple loci that reduced the CR staining and wrinkly colony morphology of this strain.

From the screen, we identified expected pathways (e.g., Pel EPS synthesis) as well as loci that have been linked previously to surface sensing. That is, in a previous study, we performed a genetic screen starting in the *P. aeruginosa* PA14 Δ*bifA* mutant background—the *bifA* gene encodes a c-di-GMP phosphodiesterase (PDE) and the Δ*bifA* mutant produces levels of c-di-GMP ~10-fold higher than the WT and cannot swarm (42, 56). We mutagenized the Δ*bifA* mutant with the mariner transposon and screened ~5,500 mutants to identify those with restored swarming motility. The list of candidates identified in that previous screen overlapped the candidate mutants identified here, either in the Δ*flgK* transposon screen or the candidate mutants we analyzed, including mutations in genes coding for the stators (*motA*), T4P-related functions linked to cAMP signaling and surface sensing (*pilY1*, *pilW*), c-di-GMP-related functions (PvrRS, a PDE/DGC pair with alleles frequently isolated as suppressors due to Mariner-based over-expression of PvrR as we previously reported), *sadB* and Pel biosynthesis (*pelA*, *pelB*, and *pelF*). This finding is perhaps not surprising given that both the Δ*bifA* and Δ*flgK* mutants have increased c-di-GMP levels. These data are consistent with the findings here that mutations in the same genes, which reduce the CR staining and wrinkly colony morphology of the Δ*flgK* mutant, also restored swarming motility to the Δ*bifA* mutant, and previous observations showing the reciprocal regulation of biofilm- versus motility-related functions (43, 71).

We and others have shown previously that T4P are key players in surface sensing (45–49, 72, 73). We note that mutations impacting the T4P were identified as suppressors of the enhanced CR binding and wrinkly colony c-di-GMP-mediated phenotypes of the Δ*flgK* mutant in the CR screen described above. We take this finding to mean that inputs from both flagella and T4P are needed to fully engage the surface sensing pathway. This conclusion is also consistent with our observation that the Δ*flgK* mutant requires surface engagement for increased levels of c-di-GMP. Interestingly, mutation of *fimV,* which strongly impacts T4P function, does not suppress the Δ*flgK* hyper-signaling phenotypes, hinting at the complexity by which the flagellar- and T4P-mediated signals may be integrated to coordinate surface sensing, which remains an open question. However, we cannot dismiss the possibility that *fimV* plays a more subtle role in this signaling pathway.

We also showed that the DGCs RoeA and SadC contribute to elevated c-di-GMP levels and enhanced signaling phenotypes of the Δ*flgK* mutant. RoeA and SadC have both been shown previously to contribute to biofilm formation, and SadC is a key component of the surface sensing pathway in *P. aeruginosa* PA14 (36, 46, 49, 56, 57). Here, we observed that mutation of the *roeA* gene exerted a stronger impact on Pel-mediated phenotypes of the Δ*flgK* mutant versus the Δ*sadC* mutation, although they both showed significant reductions in c-di-GMP levels. While the triple mutant (Δ*flgK* Δ*sadC* Δ*roeA*) is devoid of CR binding and wrinkling, the levels of c-di-GMP in this mutant are comparable to the WT (~2-fold reduction relative to Δ*flgK—*compare Fig. 3 to Fig. 1C), indicating that SadC and RoeA are largely responsible for Pel production in the Δ*flgK* mutant, but that additional DGCs are likely also contributing to global pools of c-di-GMP in this mutant background. This is not entirely surprising, given that there are at least 16 predicted DGCs encoded in the genome of *P. aeruginosa* (74).

Our results indicating RoeA plays a stronger role in impacting increased Pel production compared to SadC in the Δ*flgK* mutant agree well with data from a previous report from our group pertaining to suppression of the Δ*bifA* mutant phenotypes, with RoeA playing a stronger role in impacting increased Pel production, whereas SadC had a more robust impact on motility repression by the Δ*bifA* PDE mutant (56). In addition, BifA has recently been shown to interact directly with PelD, a c-di-GMP-binding effector of Pel production, to negatively regulate PelD function and thereby impact Pel synthesis (75, 76). Given the association of RoeA with Pel production and its genetic relationship to BifA, it is plausible that RoeA contributes positively to the c-di-GMP pools involved in activation of Pel biosynthesis. By contrast, we posit that SadC contributes to local c-di-GMP pools that repress motility, a notion supported by our previous findings that SadC interacts with the stator MotC (36). This interaction serves to stimulate SadC’s production of c-di-GMP and thus likely contributes to flagellar signaling. Given that Δ*flgK* mutants are non-motile, we cannot assess the relative contributions of SadC versus RoeA in impacting motility in this particular strain background. The cumulative evidence for differential contributions by RoeA and SadC to modulate local pools of c-di-GMP adds to a growing list of reports of DGCs and PDEs that directly interact with signaling effectors/partners to achieve localized impacts on signaling outcomes (65, 75, 77, 78), highlighting a common theme emerging for c-di-GMP signaling pathways.

It is worth noting that the difference in the strength of suppression between *roeA* and *sadC* mutation in the Δ*flgK* mutant may be one reason we isolated an allele of *roeA* but not *sadC* in the Δ*flgK* CR suppressor screen. In addition, the absence of *sadC* suppressor alleles (as well as other expected alleles such as those of *sadB*, *motAB*, and *motCD*) may be due to the screen not reaching full saturation. Finally, insertions in the *pel* locus (seven genes, *pelABCDEFG*) accounted for approximately 1 in 6 transposon mutants identified, which is not surprising given that such mutants were expected; however, a high background isolation of *pel* alleles may have precluded isolation of other non-EPS-based mutations as suppressors of the Δ*flgK* mutant. Such limitations served as the rationale for targeting candidate genes as a companion approach.

Our findings also support a role for the stators and switch complex in the phenotypes associated with the Δ*flgK* mutant. Loss of the stators and mutations that render the stators unable to conduct protons result in loss of Congo red binding and the wrinkly colony phenotype of the Δ*flgK* mutant. Regarding the *motB*(D30A) and *motD*(D23A) proton-binding mutants, our data are consistent with the notion that the ability of stators to engage in proton conductance and hence stable stator incorporation are important for enhanced signaling in the Δ*flgK* mutant. However, we cannot rule out the possibility that changes we observed, such as increased abundance of the MotD-D23A variant or the negative impact on motility by MotB D30A, alter the stoichiometry and/or dynamics of stator incorporation, and thereby indirectly affect the observed suppression of the Δ*flgK* hyper-signaling phenotypes.

In addition, we observed that mutations that impact stator interactions with the switch complex also impact CR binding and wrinkly colony appearance of the Δ*flgK* mutant. Here, we tested the stator-switch complex interaction using specific allelic combinations of *motA* and *fliG* designed to first disrupt and then restore key electrostatic interactions between these proteins. The design of these mutations was based on studies in *E. coli*. Our results are largely consistent with the notion that disruption of MotA-FliG interaction by *motA* point mutation leads to abrogation of the Δ*flgK* mutant phenotypes, and restoration of the interaction by *fliG* point mutation restores the Δ*flgK* mutant phenotypes. Unexpectedly, we observed that the *fliG*-D295A allele used in these studies enhanced the hyper-signaling phenotype in the Δ*flgK* (*motA*^+^) background, rather than the expected result of having little or no impact on the Δ*flgK* phenotypes in this strain. We interpret these observations to indicate that the FliG-D295A protein enhances interactions with the WT MotA protein, leading to increased signaling; however, further work is needed to understand the impact of this *fliG* mutation on the signaling pathway.

As mentioned earlier, a similar phenomenon was previously observed in which flagellar mutants of strain PAO1 were frequently isolated in biofilm populations as well as among cystic fibrosis sputum isolates on the basis that they exhibited a rugose small colony variant (RSCV) phenotype, characterized by overproduction of the exopolysaccharides Pel and Psl (30). In that study, Harrison et al. undertook a transposon screen to identify suppressors of the Δ*fliC* mutant RSCV phenotype. Despite a number of technical differences between our study and theirs, such as the *P. aeruginosa* strain background (PA14 vs PAO1), media type (M8 minimal medium supplemented with CR vs. Vogel Bronner minimal medium [VBMM]), genetic background (Δ*flgK* vs Δ*fliC*), and transposon used for mutagenesis (Mariner vs miniTn5Pro), there are considerable similarities in results between the two studies. For example, both studies identified suppressor mutations in a subset of T4P genes, including numerous alleles of the *pilY1* gene and alleles of the minor pilins *pilW* and *pilX*, further bolstering the notion that flagellar-mediated signaling integrates inputs from a T4P-mediated signaling arm to achieve an optimal surface recognition response. Both studies identified DGCs, with SadC in common, as well as those unique to each study (RoeA here and SiaD in their study), confirming c-di-GMP is a critical second messenger in this response. Mutations in both sets of stators were identified as suppressors in their screen and in our candidate analysis here, supporting the premise that stators are important components of flagellar-mediated surface sensing. In addition, the SadB protein, known to be involved in early surface colonization, is a common factor important for signaling in both strains. The prevalence of similarities between these studies is surprising in light of recent data showing striking differences in the surface sensing behavior of PAO1 versus PA14 cells when single-cell lineages were tracked over time during early surface colonization (79). These experimental approaches (genetic screens/macroscopic assays versus single-cell tracking/microscopy) are clearly different; however, future studies will be needed to address the reasons for such differing conclusions.

Collectively, this work, together with studies from diverse bacterial model systems noted here, clearly shows that there are conserved signaling pathways connecting the flagellum and flagellum biosynthesis to biofilm-relevant signaling. In addition, another important conclusion from these studies is the notion that bacteria monitor the status of flagellar synthesis and structural integrity as well as flagellar function, such that disruptions in flagellar biosynthesis trigger biofilm-related signaling. In general, late disruptions in flagellar synthesis (after basal body assembly, as in the case for the *fliC* and *flgK* mutants presented here and elsewhere) result in enhanced stator-dependent c-di-GMP signaling, and we believe such effects functionally mimic the surface sensing process, with cells interpreting lack of the flagellar filament as an increased load on the motor upon surface engagement. By contrast, early disruption in flagellum biosynthesis that precludes completion of basal body production generally leads to enhanced signaling in a stator-independent manner (as in the case of *fliF* mutation), indicating that bacteria utilize c-di-GMP-mediated signaling to respond to aberrant or aborted flagellar synthesis and thereby promote biofilm formation under such circumstances.

Interestingly, there is no consensus yet on whether the same DGCs are utilized to generate c-di-GMP for both stator-dependent and independent pathways or whether distinct enzymes are required. Here, we found that the same DGCs, SadC and RoeA, contribute to c-di-GMP pools in both stator-dependent and -independent (as in the case of the Δ*fliF* mutation) signaling pathways. Similar findings were reported for *V. cholera,* showing that three DGCs (CdgA*,* L, and O) largely contribute to c-di-GMP pools that impact both pathways. In *C. crescentus*, however, Hershey et al. meticulously demonstrated that flagellar disruption via the stator-independent pathway (termed “developmental pathway”) requires the DGC PleD, whereas the stator-dependent pathway (termed “mechanical pathway”) utilizes DgcB, with both pathways ultimately converging to regulate production of the holdfast adhesin responsible for surface colonization by this microbe.

**Fig 5:**
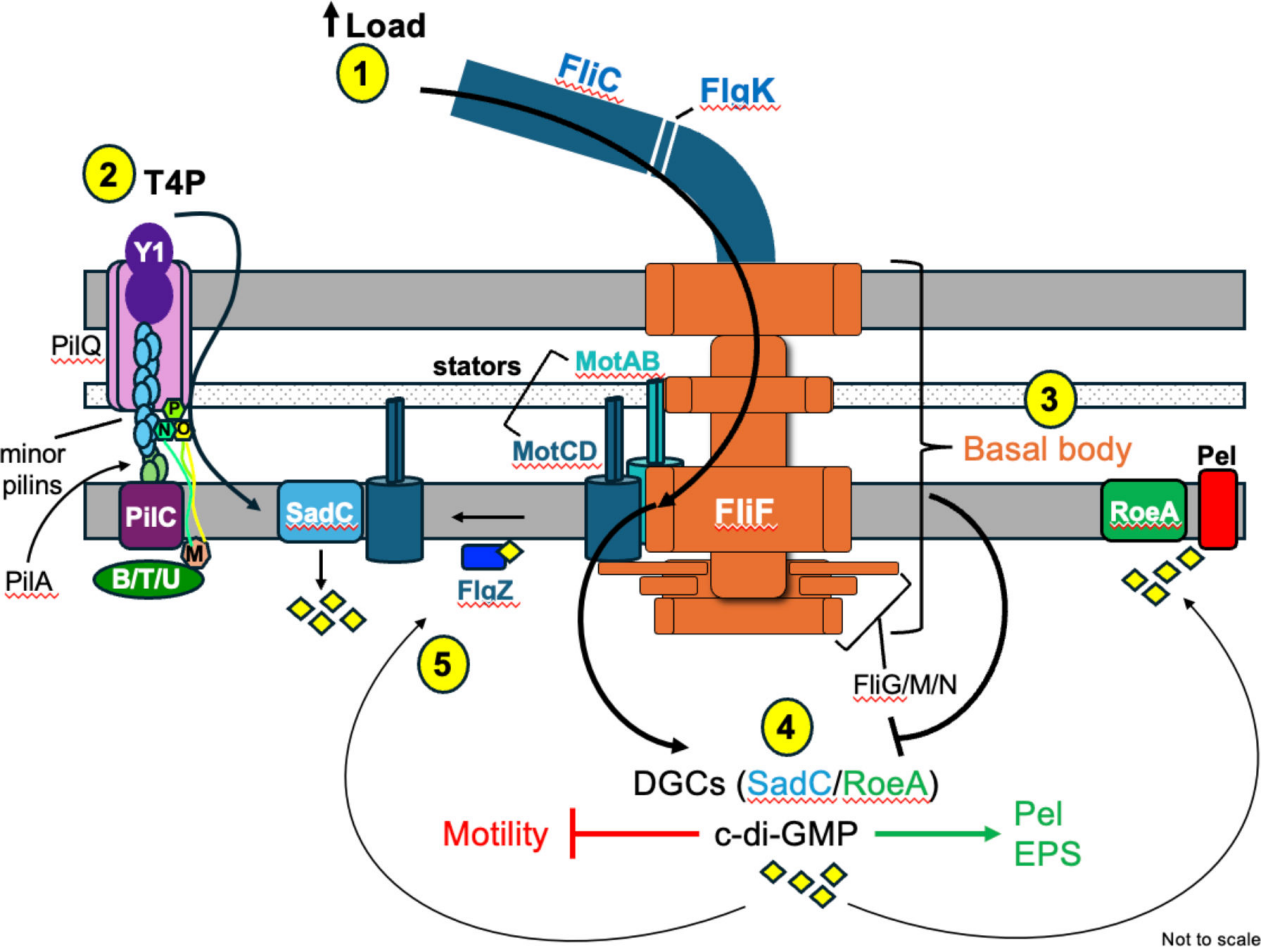
Proposed model for flagellar-mediated impacts on biofilm-relevant surface signaling. (1) Surface contact is sensed as increased load on the flagellum, which transmits this signal by modulating stator incorporation. We propose that mutations in *fliC* and *flgK* leading to loss of the flagellar filament mimic this signaling process. (2) PilY1 acts as a mechanosensor at the T4P tip to detect surface contact and relay this outside-in signal via the minor pilins (including PilW and PilX) and the alignment complex (PilMNOP) to SadC (45, 48, 49, 80). B/T/U refers to extension (PilB) and retraction ATPases (PilT/U). (3) Assembly of a complete flagellar basal body inhibits production of c-di-GMP in favor of motility. Disruption of basal body assembly elicits Pel production in a stator-independent pathway. (4) The DGCs, SadC and RoeA, contribute to pools of c-di-GMP that repress motility and activate production of Pel EPS to promote biofilm formation. (5) The FlgZ-c-di-GMP complex facilitates FlgZ-MotC interaction to remove the MotCD stator from the motor, disabling surface motility and allowing for interaction of MotC with SadC to further stimulate c-di-GMP production in a positive feedback loop (36, 37).

Together, these studies highlight the importance of understanding the role of the flagellum in surface sensing and biofilm formation and call for additional studies aimed at further exploration of this salient topic. In Fig. 5, we provide a model to summarize the findings here and integrate our data together with previous experimental data into a framework underscoring key concepts in flagellar-mediated surface sensing by *P. aeruginosa* PA14. Key questions remain regarding various aspects of the surface sensing process in *P. aeruginosa*, including: how does the stator function influence c-di-GMP production at the molecular level? What are the mechanistic features that distinguish the MotAB and MotCD stator sets and their roles in transducing the flagellar surface sensing signal? What additional regulatory proteins or pathways might modulate stator-mediated signaling? These and related questions are the focus of current and future work from our group.

## MATERIALS AND METHODS

### Strains and media

*P. aeruginosa* UCBPP PA14 was used as the WT strain, and all mutations were made in this background unless stated otherwise. Mutations were made using *E. coli* S17-1 λpir for conjugation with PA14. All strains used in this study are listed in Table S2. Bacterial strains were cultured in 5 mL of lysogeny broth (LB) medium or plated on 1.5% LB agar with antibiotics, when necessary. Gentamicin (Gm) was used at 25 or 30 µg/mL for *P. aeruginosa* and 10 µg/mL for *E. coli*. Carbenicillin (Cb) was used at 100 µg/mL for *E. coli,* and triclosan was used at 20 µg/mL for counter-selection against *E. coli* after conjugation with *P. aeruginosa*. M8 minimal salts medium supplemented with MgSO_4_ (1 mM), glucose (0.2%), and casamino acids (0.5%) was used for all assay conditions (81).

### Construction of mutant strains and plasmids

Plasmids used in this study are listed in Table S3 and primers used in this study are listed in Table S4. Plasmids to generate gene deletions and point mutations were generated by PCR and Gibson assembly (82) and cloned into the pMQ30 vector (83). In-frame deletions and point mutants were generated using allelic exchange as previously described (83, 84).

### Transposon mutagenesis and identification of integration site

Transposon mutants were generated with the Mariner transposon as previously described (85). Briefly, *E. coli* S17 harboring the pBT20 plasmid harboring the Mariner transposon was co-incubated with *P. aeruginosa* PA14 ∆*flgK* on LB agar for 1 hour at 30°C for conjugation to occur. Cells were then scraped up, diluted, and plated on LB agar plates supplemented with 30 µg/mL Gm, 20 µg/mL triclosan, 0.04 mg/mL Congo Red, and 0.01 mg/mL Coomassie blue. Plates were then incubated at 37°C for 24 hours and then at room temperature for 48 hours. Colonies that displayed altered colony morphology or Congo Red uptake relative to the ∆*flgK* strain were selected and confirmed with a second round of plating on Congo Red agar with selection. After confirmation of the phenotype, arbitrary primed PCR was then performed and sequenced using the Sanger method to identify the location and direction of the transposon as previously described (7).

### Congo Red assay

Congo Red dye uptake was adapted from previously published protocols (38). Briefly, M8 agar (1%) plates supplemented with Congo Red solution (final concentration CR at 0.04 mg/mL with 0.01 mg/mL Coomassie blue) were spotted with 2 μL of an overnight culture and incubated at 37°C for 16 hours and then at room temperature for an additional 4 days.

### Swimming motility assay

Swimming assays were performed as previously described (86). M8 medium was supplemented with 0.3% agar. Swim plates were inoculated with sterile tips dipped into an overnight culture and incubated at 37°C for 16–18 hours.

### Swarming motility assay

Swarming assays were performed as previously described (87). M8 medium was supplemented with 0.5% agar. Swarm plates were inoculated with 2 μL of an overnight culture and incubated at 37°C for 16 hours.

### Twitching motility assay

M8 medium was supplemented with 1% agar. Plates were inoculated with sterile tips dipped into an overnight culture and stabbed through the agar to the plate bottom and incubated at 37°C for 16–18 hours, followed by 5 days at room temperature.

### Protein detection and quantification

Cells were harvested from swarm plates grown for 16 h at 37°C, normalized by OD_600_, and lysed by boiling in SDS gel loading buffer (50 mM Tris-HCl pH 6.8, 2% SDS, 10% glycerol, 0.1% bromophenol blue, and supplemented with freshly added DTT at a final concentration of 100 mM). Equal volumes of sample lysates were then resolved using 10% SDS-PAGE TGX gels (Bio-Rad, Hercules, CA). Proteins were transferred to a nitrocellulose membrane using a Trans-Blot Turbo system (Bio-Rad, Hercules, CA) and probed with anti-His antiserum (Qiagen, Germantown, MD) or anti-GFP antiserum (BioLegend, San Diego, CA) at a 1:2,000 dilution prepared in 1× TBS, 3% BSA. Proteins were detected using fluorescence detection with IRDye-labeled fluorescent secondary antibodies and imaged using the Odyssey CLx Imager (LICOR Biosciences, Inc., Lincoln, NE). Image Studio Lite software (LICOR Biosciences, Inc., Lincoln, NE) was used to quantify protein levels using a non-specific band present in all lanes as a normalization control. During Western blotting of the MotB WT and D30A variant, we noticed a change in mobility of the D30A variant protein relative to the WT protein. This mobility shift was observed in both the Δ*flgK* mutant (shown in Fig. 2A) and in the WT background (Fig. S3C). One possible explanation for a size shift is a change in the MotB DNA coding sequence, such as a DNA insertion or a mutation of the stop codon leading to read-through into a downstream sequence. We investigated these possibilities by sequencing a PCR product amplified from the genomic region encompassing the *motB* gene as well as upstream and downstream sequences in both the WT and variant *motB* genes in the WT and Δ*flgK* strains; however, we did not find any mutations consistent with this explanation. An alternative possibility that has yet to be explored is that the MotB-D30A protein undergoes post-translational modification that alters its gel migration.

### Cellular fractionation

Bacterial cells were collected from swarm plates grown for 16 h at 37°C using plastic coverslips, and cells were harvested by centrifugation at 15,870 × *g* for 5 min at room temperature. Cell pellets were frozen at −80°C and then resuspended in lysis buffer (50 mM Tris-HCl [pH 8.0], 1 mM EDTA, 2 mM MgCl_2_) supplemented with protease inhibitors (Pierce Biotechnology, Waltham, MA), followed by treatment with lysozyme (final concentration 0.1 mg/mL) (RPI, Mount Prospect, IL) and Benzonase nuclease at a final concentration of ~50 units/mL (Novagen, San Diego, CA) for 15 min at RT with inversion mixing. Cells were then lysed by sonication (30% amplitude for 10 s, 1–2 times) on ice. Samples were centrifuged at 9,300 × *g* for 5 min at 4°C to remove unbroken cells, and supernatants were collected as whole-cell lysates. Samples were further fractionated to isolate the total membrane and cytoplasmic fractions, as described previously (35). Concentrations of total protein in each fraction were determined using a BCA protein assay kit (Pierce Biotechnology, Waltham, MA). For Western blots of cellular fractions, samples from each fraction were normalized to the same concentration, and equivalent amounts of total protein were mixed with a 2× SDS loading buffer containing freshly added dithiothreitol (200 mM). Samples were heated at 70°C for 5 minutes to denature proteins and then resolved as described in the Protein Detection method section above.

### Protein sequence alignments

Pairwise alignments for MotA of *P. aeruginosa* strain PA14 (encoded by *PA14_65450*) and *E. coli* K12 (encoded by *b1890*) and for FliG of *P. aeruginosa* (encoded by *PA14_50130*) and *E. coli* (encoded by *b1939*) were generated using the EMBOSS Needle tool (https://www.ebi.ac.uk/jdispatcher/psa/emboss_needle).

The multiple sequence alignment of MotB from *E. coli* (*b1889*) and *P. aeruginosa* (*PA14_65430*) and MotD (PA14_45540) was generated using the MUSCLE tool (https://www.ebi.ac.uk/jdispatcher/msa/muscle?stype=protein). We note here that in a previous report (35), we used the *E. coli* nomenclature to refer to the analogous *P. aeruginosa* MotA and FliG amino acid residues, whereas here we referred to those same amino acids using the *P. aeruginosa* amino acid numbers according to the alignments shown in Fig. S2.

### Cyclic-di-GMP quantification

c-di-GMP levels were quantified via liquid chromatography-mass spectrometry (LC-MS) at the Michigan State University Mass Spectrometry and Metabolomics Core. For surface-grown c-di-GMP measurements, cells were harvested from swarm plates after ~16 hours of growth as previously reported (44, 48). For liquid-grown c-di-GMP measurements, cells were sub-cultured (1:100) from an LB-grown overnight culture in M8 liquid media and harvested after 3 hours at 37°C, whereupon cultures were normalized to an OD_600_ of 0.4 prior to nucleotide extraction as described (44). Measurements were normalized to the dry weight of cell pellets after nucleotide extraction. All experiments were performed in triplicate with three technical replicates per strain.

### Statistical methods

Data were analyzed by ordinary one-way ANOVA with a post hoc test for multiple comparisons (the specific test used for each case is identified in the figure legend) or using unpaired t-test using GraphPad Prism software (La Jolla, CA).

## SUPPLEMENTAL MATERIAL

Supplemental figures jb.00520-24-s0001.pdf Fig. S1 to S5.

Supplemental tables jb.00520-24-s0002.pdf Tables S1 to S4.

ASM does not own the copyrights to Supplemental Material that may be linked to, or accessed through, an article. The authors have granted ASM a non-exclusive, world-wide license to publish the Supplemental Material files. Please contact the corresponding author directly for reuse.
