# Supplementary material for: Genetic analysis of flagellar-mediated surface sensing by Pseudomonas aeruginosa PA14
Source: J Bacteriol. 2025 Jun 5;207(7):e00520-24. doi: 10.1128/jb.00520-24 (PMC12288467; doi:10.1128/jb.00520-24)
Supplement: Supplemental figures — Fig. S1 to S5. [file jb.00520-24-s0001.pdf]

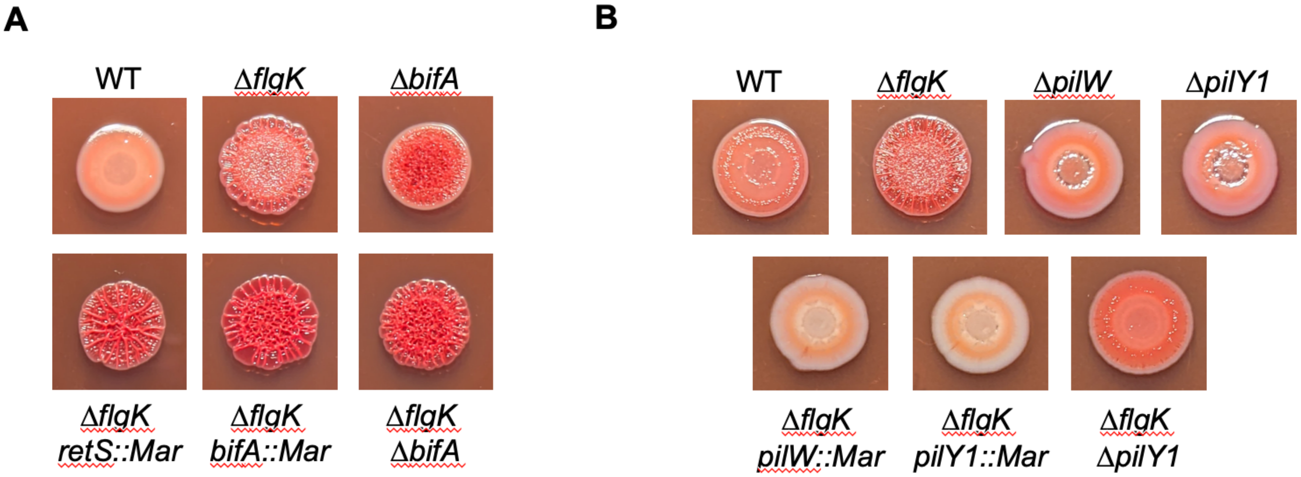

Figure S1. Representative enhancers (A) and suppressors (B) of the Δ*flgK* CR and wrinkled colony phenotypes isolated by transposon mutagenesis. Representative CR images of the indicated strains. Images of the mutants in **A** were taken at 4 days post-inoculation compared to the typical 5 days, to best capture the enhanced CR phenotype of the mutants shown in the bottom row compared to the single Δ*flgK* mutant (top middle).

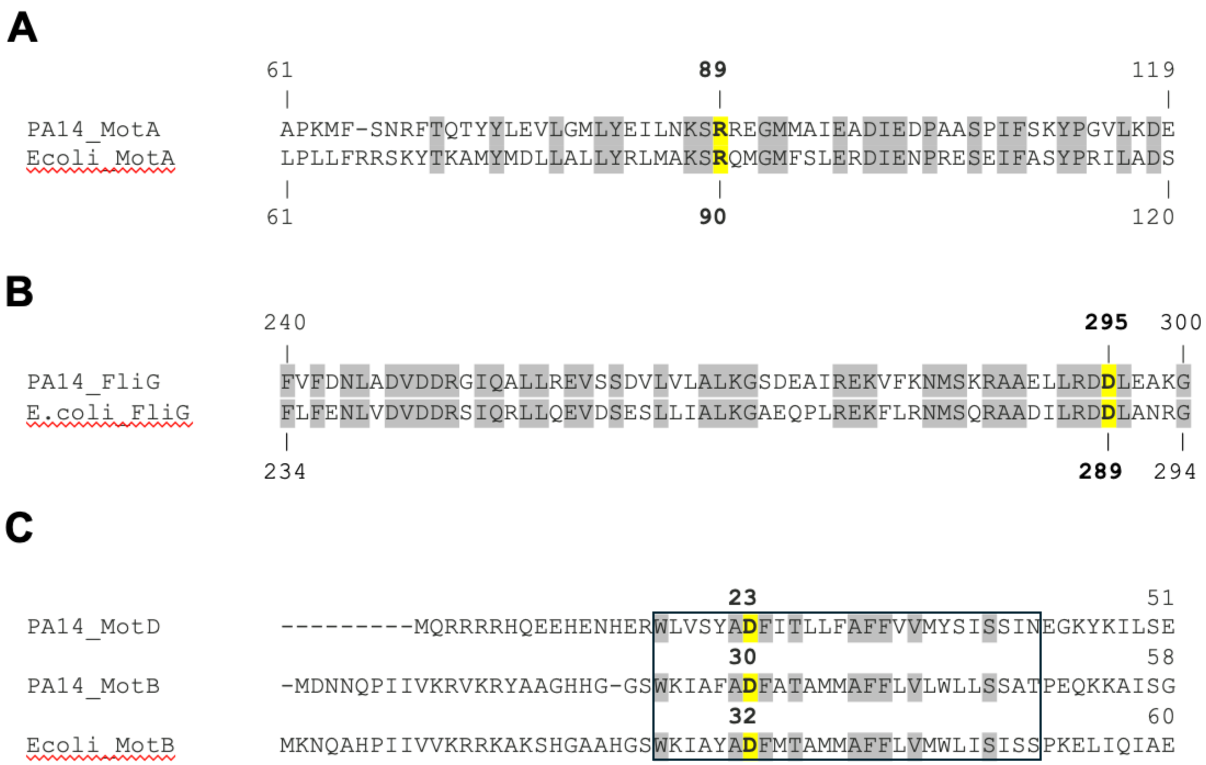

Figure S2. Sequence alignments of *P. aeruginosa* and *E. coli* flagellar proteins. Pairwise alignments of **A.** MotA of *P. aeruginosa* strain PA14 (encoded by *PA14_65450*) and *E. coli* K12 (encoded by *b1890*) and **B.** FliG of *P. aeruginosa* (*PA14_50130*) and *E. coli* (*b1939*) were generated using the EMBOSS Needle tool (https://www.ebi.ac.uk/jdispatcher/psa/emboss_needle). Full protein sequences were aligned and relevant regions of those alignments are shown. Gray boxes indicate conserved residues. Amino acids in bold with yellow highlighting indicate conserved residues mutated for this study. **C.** Multiple sequence alignment of MotB from *E. coli* (*b1889*) and *P. aeruginosa* (*PA14_65430*) and MotD (*PA14_45540*) was generated using the MUSCLE tool (https://www.ebi.ac.uk/jdispatcher/msa/muscle?stype=protein). Boxed region indicates the predicted transmembrane region including the conserved proton-binding Aspartate residues in bold with yellow highlighting.

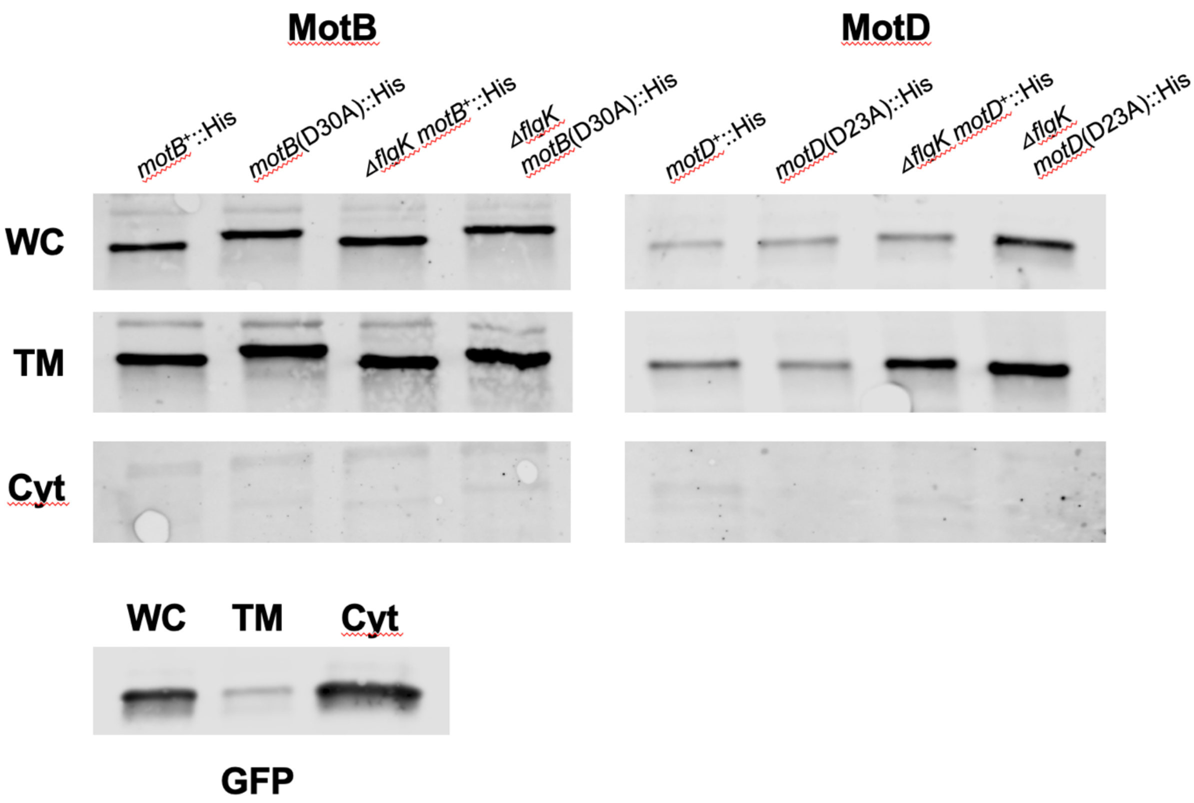

Figure S3. MotB-D30A and MotD-23A variant proteins localize to the total membranes of fractionated cells. Representative Western blots of indicated strains expressing His_6-epitope tagged WT MotB and the D30A variant (left panels) and WT MotD and D23A variant (right panels) in whole cell (WC, top), total membrane (TM, middle) and cytoplasmic (Cyt, bottom) cellular fractions. The bottom most panel shows representative fractions of a WT strain expressing GFP, as a control for cytoplasmic and membrane fractionation. Bacterial cells were collected from swarm plates grown for 16h at 37°C, lysed by sonication and fractionated into WC and Cyt and TM subcellular fractions. Equal amounts of total protein were resolved using SDS-PAGE and proteins were detected using either anti-His or anti-GFP antisera followed by fluorescence

detection with IRDye-labeled fluorescent secondary antibodies and imaged using the Odyssey CLx Imager.

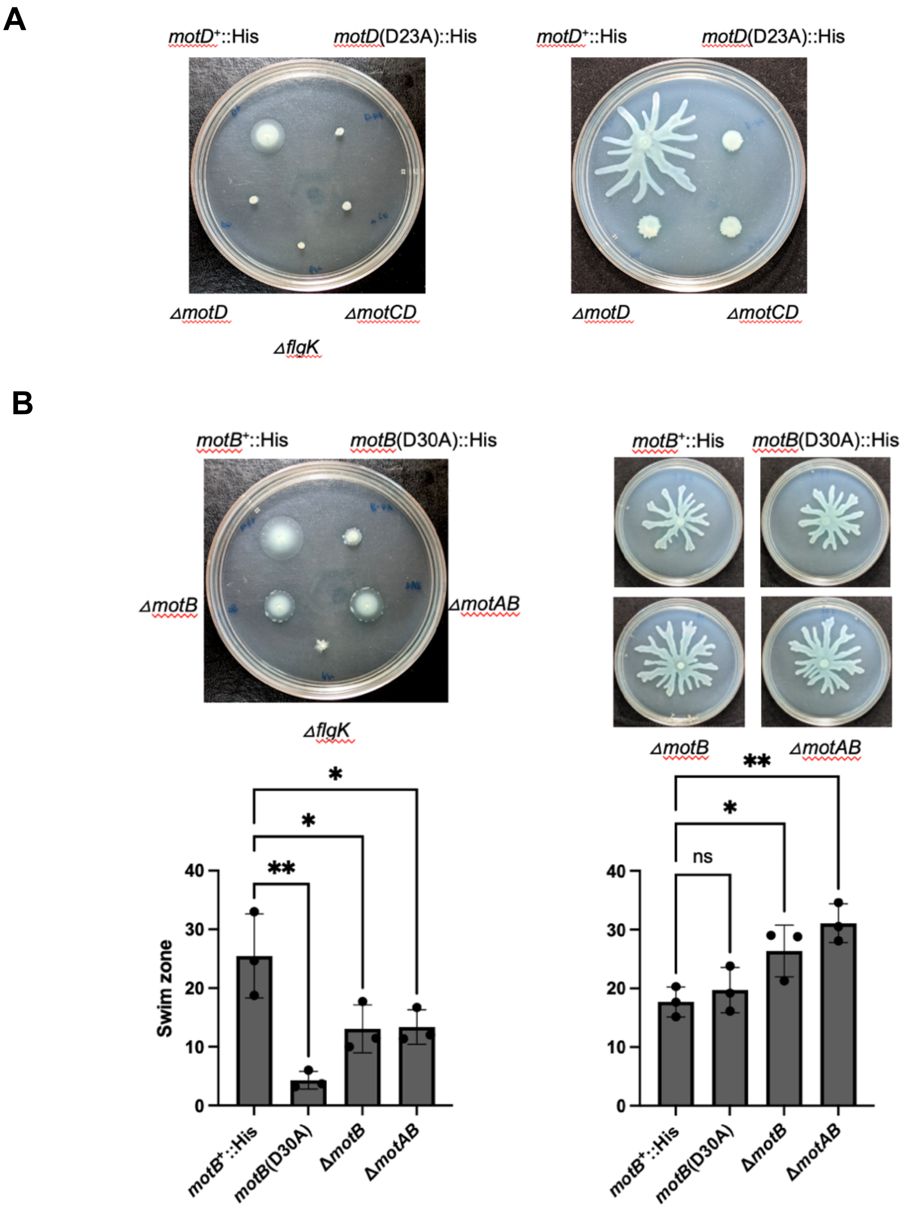

Figure S4. Motility assays of the *motB*(D30A) and *motD*(D23A) proton binding mutants. Swimming (left) and swarming (right) assays of the indicated *motD* mutant strains (**A)** and *motB* mutant strains (**B)**. Swim plates (0.3% agar) and swarm plates (0.5% agar) were incubated at 37° C for 16. Graphs show quantification of swimming (left) and swarming (right) for the indicated *motB* mutant strains. Motility zones were

measured using ImageJ. Motility experiments were performed in triplicate with three technical replicates per strain and analyzed by ANOVA with Dunnett's multiple comparisons test. Significant differences are shown for comparisons to the *motB*$^+$::His strain. ns, non-significant difference; *, $P < 0.05$ and **, $P < 0.01$.

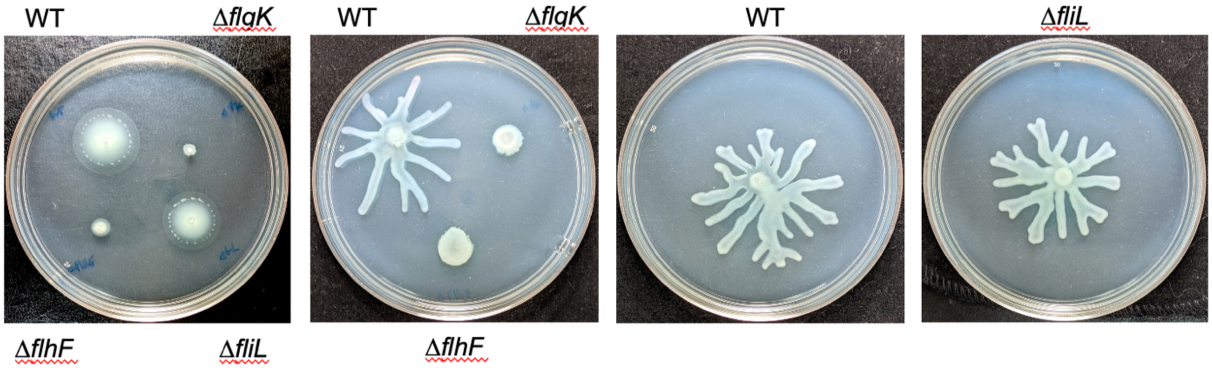

Figure S5. Motility assays of the Δ*flhF* and Δ*fliL* mutants. Representative images of swimming (left image) and swarming (right three images) assays of the indicated WT and mutant strains.
