# Supplementary material for: Genetic analysis of flagellar-mediated surface sensing by Pseudomonas aeruginosa PA14
Source: J Bacteriol. 2025 Jun 5;207(7):e00520-24. doi: 10.1128/jb.00520-24 (PMC12288467; doi:10.1128/jb.00520-24)
Supplement: Supplemental tables — Tables S1 to S4. [file jb.00520-24-s0002.pdf]

Table S1. Mutations identified from Congo red transposon screen in *flgK* mutant background with no known association with EPS production and/or c-di-GMP signaling.

Candidate[a]	Predicted or known function	Congo red phenotype[b]	Number of alleles isolated
**Redox-related functions**			
speA	arginine dearboxylase	Reduced	1
sodM	superoxide dismutase	Enhanced	1
katA	catalase	Enhanced	1
PA14_44350	cbb3-type cytochrome c oxidase subunit II	Enhanced	1
PA14_57570	cytochrome c reductase	Enhanced	3
**Regulators**			
PA14_16550	Putative transcriptional regulator	Reduced	1
PA14_43670	Histidine kinase, part of a two-component system	Reduced	1
hflX	Role in lysogeny	Reduced	1
aguR	Transcription factor, negative regulation of hydrolase activity	Enhanced	1
**Other functions**			
PA14_11290	Putative permease – membrane transport proteins	Reduced	1
thdF	Putative GTP binding protein - GTPase	Reduced	1
ppK	Polyphosphate kinase – responsible for the synthesis of inorganic polyphosphate from ATP	Reduced	1
ptsP	phosphoenolpyruvate protein phosphotransferase, downstream gene (PA14_04420) has PAS/GGDEF domain	Enhanced	1
hepP	heparanase	Reduced	1
PA14_30470	periplasmic aliphatic sulfonate binding protein	Reduced	1
PA14_02890	nucleoside channel forming protein	Reduced	1
PA14_72870	aminotransferase, biosynthesis of secondary metabolites	Enhanced	1
PA14_08600	23S rRNA,	Enhanced	1
PA14_08570	16S rRNA	Enhanced	1
orfN	NAD-dependent epimerase/dehydrase, glycosylation, group 4 glycosyl transferase	Reduced	1
PA14_08580	tRNA-Ile	Enhanced	1
PA14_40660	T6SS effector Tse1, amidase activity	Yellow, smooth	1
PA14_32820	PA2462 homolog of PAO1	Enhanced	1
PA14_70870	5s rRNA	Enhanced	1
PA14_30100	50S ribosomal protein L16 3-hydroxylase	Enhanced	1
PA14_66100	O-antigen ligase, WaaL, critical for cell wall integrity and motility	Enhanced	1
purM	phosphoribosylaminoimidazole	Enhanced	1
PA14_41280	beta-lactamase	Enhanced	1

[a]The source of the gene information is Pseudomonas.com.
[b]"Reduced" CR phenotype indicates reduction in both red color binding and wrinkled colony morphology, unless otherwise indicated. "Enhanced" CR phenotype indicates additional red color/wrinkling.

Supplementary Table S2. Strains used in this study.

Strain name	Relevant genotype and description	Source
***E. coli* strains**		
DH5α	*supE*44 Δ*lacU*169(φ80*lacZ*ΔM15) *hsdR*17 *thi*-1 *relA*1 *recA*1	Life Technologies
S17-1 (λpir)	*thi pro hsdR*- *hsdM*+ Δ*recA* RP4-2::TcMu-Km::Tn7	(1)
***P. aeruginosa* strains (SMC#)**		
232	PA14 wild type (WT)	(2)
9002	*flgK* deletion mutant (Δ*flgK*)	This study
7596	*fliC* deletion mutant (Δ*fliC*)	(3)
2893	Δ*pelA*	(4)
7297	Δ*flgK* Δ*pelA*	(3)
10227	Δ*fliC* Δ*pelA*	This study
3351	Δ*bifA*	(5)
6592	Δ*bifA* Δ*flgK*	This study
3718	Δ*pilY1*	(6)
9212	Δ*flgK* Δ*pilY1*	This study
4020	Δ*pilW*	(7)
9202	Δ*flgK* Δ*motAB*	This study
9203	Δ*flgK* Δ*motCD*	This study
9204	Δ*flgK* Δ*motABCD*	This study
2385	PA14 wild type + pSMC21	(8)
5797	*motA*(R89E)::His_6	(9)
9208	Δ*flgK* *motA*(R89E)::His_6	This study
6009	*motA*::His_6 *fliG* (D295A)	This study
9209	Δ*flgK* *motA*::His_6 *fliG* (D295A)	This study

10160	Δ*flgK motA*(R89E)::His_6 *fliG* (D295A)	This study
10159	*motA*(R89E)::His_6 *fliG* (D295A)	This study
9574	*motB*$^+$::His_6	This study
9575	Δ*flgK motB*$^+$::His_6	This study
9572	Δ*flgK motB* (D30A)::His_6	This study
9577	*motD*$^+$::His_6	This study
9578	Δ*flgK motD*$^+$::His_6	This study
9560	Δ*flgK motD*(D23A)::His_6	This study
6591	Δ*flgK* Δ*sadC*	This study
6593	Δ*flgK* Δ*roeA*	This study
6594	Δ*flgK* Δ*sadC* Δ*roeA*	This study
9543	Δ*fliL*	This study
9544	Δ*flgK* Δ*fliL*	This study
9540	Δ*flhF*	This study
10240	Δ*flhF* Δ*motABCD*	This study
9541	Δ*flgK* Δ*flhF*	This study
9715	Δ*fliF*	This study
9775	Δ*fliF* Δ*motAB*	This study
9776	Δ*fliF* Δ*motCD*	This study
10241	Δ*fliF* Δ*sadC*	This study
10242	Δ*fliF* Δ*roeA*	This study
9757	Δ*fliG*	This study
10239	Δ*fliG* Δ*motABCD*	This study
9756	Δ*fliMN*	This study
10238	Δ*fliMN* Δ*motABCD*	This study
6726	Δ*fimV*	(10)

9713	Δ*flgK* Δ*fimV*	This study
10201	Δ*fimW*	This study
10202	Δ*flgK* Δ*fimW*	This study
9771	Δ*sadB*	This study
9772	Δ*flgK* Δ*sadB*	This study

Supplementary Table S3. Plasmids used in this study.

Plasmid name	Description	Source
pBT20	Vector carrying Mariner transposon; Ap^r (backbone) Gm^r (on transposon)	(11)
pMQ30	Shuttle vector for yeast cloning and Gram-negative allelic replacement; Gm^r	(12)
pMQ30-*flgK*-KO	Plasmid for deletion of *flgK* gene; Gm^r	This study
pMQ30-*pelA*-KO	Plasmid for deletion of *pelA* gene; Gm^r	(13)
pMQ-30-*motB*(D30A)-KI	Plasmid for chromosomal substitution of D30A in *motB* gene; Gm^r	This study
pMQ-30-*motB*(D23A)-KI	Plasmid for chromosomal substitution of D23A in *motD* gene; Gm^r	This study
pSMC21	pUCP-based plasmid containing *gfpmut2*, Ap^r , Cb^r , Km^r	(14)
pMQ-30-*fliL*-KO	Plasmid for deletion of *fliL* gene; Gm^r	This study
pMQ-30-*flhF*-KO	Plasmid for deletion of *flhF* gene; Gm^r	This study
pMQ-30-*fliF*-KO	Plasmid for deletion of *fliF* gene; Gm^r	This study
pMQ-30-*fliG*-KO	Plasmid for deletion of *fliG* gene; Gm^r	This study
pMQ-30-*fliMN*-KO	Plasmid for deletion of *fliMN* genes; Gm^r	This study
pMQ-30-*fimW*-KO	Plasmid for deletion of *fimW* gene; Gm^r	This study
pMQ-30-*fimV*-KO	Plasmid for deletion of *fimV* gene; Gm^r	(10)
pMQ-30-*sadB*-KO	Plasmid for deletion of *sadB* gene; Gm^r	This study

Supplementary Table S4. Primers used in this study.

Primer name	Primer sequence (5'-3')[a]
motB KI 5'	aagcttgcatgcctgcaggtcgactGTCTGTCATGGGTGTCG
motB KI 3'	ttcgagctcggtacccggggatcctCAAACGCCACTTGACC
motB(D30A)-KI	CTCGTGGAAGATCGCCTTCGCC**GCA**TTCGCGACGGCGATGATGGCG
motB(D30A)-KI	CATCATCGCCGTCGCGAA**TGC**GGCGAAGGCGATCTTCCAC
motD KI 5'	aagcttgcatgcctgcaggtcgactGCAGGGCGGCAGCCAGGTGTG
motD KI 3'	ttcgagctcggtacccggggatcctGACTGCAAGGCCAGTTGCGAAC
motD(D23A)-KI F	CTGGCTGGTTTCCTATGCC**GCT**TTCATCACCCTGTTGTTCG
motD(D23A)-KI R	CGAACAACAGGGTGATGAA**AGC**GGCATAGGAAACCAGCCAG
fliL-KO-1	tgtaaaacgacggccagtgccaagcttgcatgcctgGTGAACATGGCCTTGGAGCAG
fliL-KO-2	*GATAGCCAGTTGGCCGGTTTC*AGCCATGCCAAAAATCCGTCG
fliL-KO-3	CGACGGATTTTTGGCATGGCT*GAAACCGGCCAACTGGCTATC*
fliL-KO-4	ccatgattacgaattcgagctcggtacccggggatccACCGCTTCGAGAATCTGTAGC
flhF-KO-1	tgtaaaacgacggccagtgccaagcttgcatgcctgCTTCCTGATGCCCTCGGTG
flhF-KO-2	CGTCCTCGCTGGGCTCTTC*CAGTTCGTCGCGGACCAG*
flhF-KO-3	*CTGGTCCGCGACGAACTG*GAAGAGCCCAGCGAGGACG
flhF-KO-4	ccatgattacgaattcgagctcggtacccggggatccCGACGAAGAACTCCAGGTGC
fliF-KO-1	aagcttgcatgcctgcaggtcgactCTGCCGATAACCGGAGCAGG
fliF-KO-2	GACGATTCTCACTCATGGCGC*CGAACTAGTTATCCTCGCGC*
fliF-KO-3	*GCGCGAGGATAACTAGTTCG*GCGCCATGAGTGAGAATCGTC
fliF-KO-4	ttcgagctcggtacccggggatcctCGAGGAGACGCGCAGGACGATATC
fliG-KO-1	aagcttgcatgcctgcaggtcgactTATCCTGCAACCTGTGCTGG
fliG-KO-2	GCACCTTCCACTTCGCTGAC*CTGCACCTCTTTCGGTCCCATG*
fliG-KO-3	*CATGGGACCGAAAGAGGTGCAG*GTCAGCGAAGTGGAAGGTGC
fliG-KO-4	ttcgagctcggtacccggggatcctCATCAGGTACACCTTGCTACCG

fliMN-KO-1	aagcttgcatgcctgcaggtcgactCCAGCAGGGCATGAACCAG
fliMN-KO-2	*GCGATCAGGGTGCCGTTGA*CGCCTCGACTTCGGTTTCC
fliMN-KO-3	GGAAACCGAAGTCGAGGCG*TCAACGGCACCCTGATCGC*
fliMN-KO-4	ttcgagctcggtacccggggatcctGCACGAACAGCTCCAGGTC
fimW-KO-1	aagcttgcatgcctgcaggtcgactTGCGCGAGATGGTCTACGTTC
fimW-KO-2	*CTGTGACCGGCTTATCGCT*CGCTTCCTGGACCACGATCAG
fimW-KO-3	CTGATCGTGGTCCAGGAAGCG*AGCGATAAGCCGGTCACAG*
fimW-KO-4	ttcgagctcggtacccggggatcctAGCGAGGGGTAGCCGTTGAG
sadB-KO-1	aagcttgcatgcctgcaggtcgactGCCTTGCACTGGCGATCATC
sadB-KO-2	CTTCCGCTTCCAGCACCTTGAGCAGCTGGAGGATGACCTG
sadB-KO-3	CAGGTCATCCTCCAGCTGCTCAAGGTGCTGGAAGCGGAAG
sadB-KO-4	ttcgagctcggtacccggggatcctGGGAAGACAGCACGGACAC

[a] In primer sequences, lower case text indicates sequence complementary to the cloning vector, upper case text indicates *Pseudomonas*-specific genomic sequence, upper case italicized text indicate 5’ sequence flanking a deletion mutation, upper case underlined text signifies 3’ sequence flanking a deletion mutation and upper case bold text indicate sequences of point mutations with the sequence of the codon underlined. Primer sequences used for sequencing the above constructs are available by request.

Literature Cited.

1. Simon, R., Priefer, U., Pühler, A. 1983. A broad host range mobilization system for in vivo genetic engineering: transposon mutagenesis in Gram negative bacteria. Bio/Technology 1:784–791.

2. Rahme LG, Stevens EJ, Wolfort SF, Shao J, Tompkins RG, Ausubel FM. 1995. Common virulence factors for bacterial pathogenicity in plants and animals. Science 268:1899–1902.

3. Lewis KA, Vermilyea DM, Webster SS, Geiger CJ, de Anda J, Wong GCL, O'Toole GA, Hogan DA. 2022. Nonmotile subpopulations of *Pseudomonas aeruginosa* repress flagellar motility in motile cells through a type IV Pilus-and Pel-Dependent mechanism. J Bacteriol 204:e00528-21.

4. Friedman L, Kolter R. 2004. Genes involved in matrix formation in *Pseudomonas aeruginosa* PA14 biofilms. Mol Microbiol 51:675–690.

5. Kuchma SL, Brothers KM, Merritt JH, Liberati NT, Ausubel FM, O'Toole GA. 2007. BifA, a cyclic-di-GMP phosphodiesterase, inversely regulates biofilm formation and swarming motility by *Pseudomonas aeruginosa* PA14. J Bacteriol 189:8165–78.

6. Kuchma SL, Ballok AE, Merritt JH, Hammond JH, Lu W, Rabinowitz JD, O'Toole GA. 2010. Cyclic-di-GMP-mediated repression of swarming motility by *Pseudomonas aeruginosa*: The *pilY1* gene and its impact on surface-associated behaviors. J Bacteriol 192:2950–64.

7. Kuchma SL, Griffin EF, O'Toole GA. 2012. Minor pilins of the type IV pilus system participate in the negative regulation of swarming motility. J Bacteriol 194:5388–403.

8. Kuchma SL, Connolly JP, O'Toole GA. 2005. A three-component regulatory system regulates biofilm maturation and type III secretion in *Pseudomonas aeruginosa*. J Bacteriol 187:1441–1454.

9. Kuchma SL, Delalez NJ, Filkins LM, Snavely EA, Armitage JP, O'Toole GA. 2015. Cyclic di-GMP-mediated repression of swarming motility by *Pseudomonas aeruginosa* PA14 requires the MotAB stator. J Bacteriol 197:420–430.

10. Luo Y, Zhao K, Baker AE, Kuchma SL, Coggan KA, Wolfgang MC, Wong GCL, O'Toole GA. 2015. A hierarchical cascade of second messengers regulates *Pseudomonas aeruginosa* surface behaviors. MBio 6:e02456-14.

11. Kulasekara HD, Ventre I, Kulasekara BR, Lazdunski A, Filloux A, Lory S. 2005. A novel two-component system controls the expression of *Pseudomonas aeruginosa* fimbrial *cup* genes. Mol Microbiol 55:368–380.

12. Shanks RMQ, Caiazza NC, Hinsa SM, Toutain CM, O'Toole GA. 2006. *Saccharomyces cerevisiae*-based molecular tool kit for manipulation of genes from Gram-negative bacteria. Appl Environ Microbiol 72:5027–5036.

13. Baker AE, Diepold A, Kuchma SL, Scott JE, Ha DG, Orazi G, Armitage JP, O'Toole GA. 2016. PilZ domain protein FlgZ mediates cyclic di-GMP-dependent swarming motility control in *Pseudomonas aeruginosa*. J Bacteriol 198:1837–1846.

14. Bloemberg GV, O'Toole GA, Lugtenberg BJ, Kolter R. 1997. Green fluorescent protein as a marker for *Pseudomonas spp*. Appl Environ Microbiol 63:4543–4551.
